# *MicroRNA-98*: the multifaceted regulator in human cancer progression and therapy

**DOI:** 10.1186/s12935-024-03386-2

**Published:** 2024-06-13

**Authors:** Vajihe Hazari, Sahar Ahmad Samali, Payam Izadpanahi, Homa Mollaei, Farzad Sadri, Zohreh Rezaei

**Affiliations:** 1 Department of Obstetrics and Gynecology, School of Medicine, Rooyesh Infertility Center, Birjand University of Medical Sciences, Birjand, Iran; 2 Department of Microbiology, Yasooj Branch, Islamic Azad University, Yasooj, Iran; 3 Reza Radiotherapy and Oncology Center, Mashhad, Iran; 4 Department of Biology, Faculty of Sciences, University of Birjand, Birjand, Iran; 5 Student Research Committee, Birjand University of Medical Sciences, Birjand, Iran; 6 Department of Biology, University of Sistan and Baluchestan, Zahedan, Iran; 7 Cellular and Molecular Research Center, Birjand University of Medical Sciences, Birjand, Iran

**Keywords:** *MicroRNA-98*, Human cancers, Oncogenic, Tumor-suppressive, And biomarkers

## Abstract

*MicroRNA-98* (*miR-98*) stands as an important molecule in the intricate landscape of oncology. As a subset of microRNAs, these small non-coding RNAs have accompanied a new era in cancer research, underpinning their significant roles in tumorigenesis, metastasis, and therapeutic interventions. This review provides a comprehensive insight into the biogenesis, molecular properties, and physiological undertakings of *miR-98*, highlighting its double-edged role in cancer progression—acting both as a tumor promoter and suppressor. Intriguingly, *miR-98* has profound implications for various aspects of cancer progression, modulating key cellular functions, including proliferation, apoptosis, and the cell cycle. Given its expression patterns, the potential of *miR-98* as a diagnostic and prognostic biomarker, especially in liquid biopsies and tumor tissues, is explored, emphasizing the hurdles in translating these findings clinically. The review concludes by evaluating therapeutic avenues to modulate *miR-98* expression, addressing the challenges in therapy resistance, and assessing the efficacy of *miR-98* interventions. In conclusion, while *miR-98*’s involvement in cancer showcases promising diagnostic and therapeutic avenues, future research should pivot towards understanding its role in tumor-stroma interactions, immune modulation, and metabolic regulation, thereby unlocking novel strategies for cancer management.

**Graphical Abstract:**

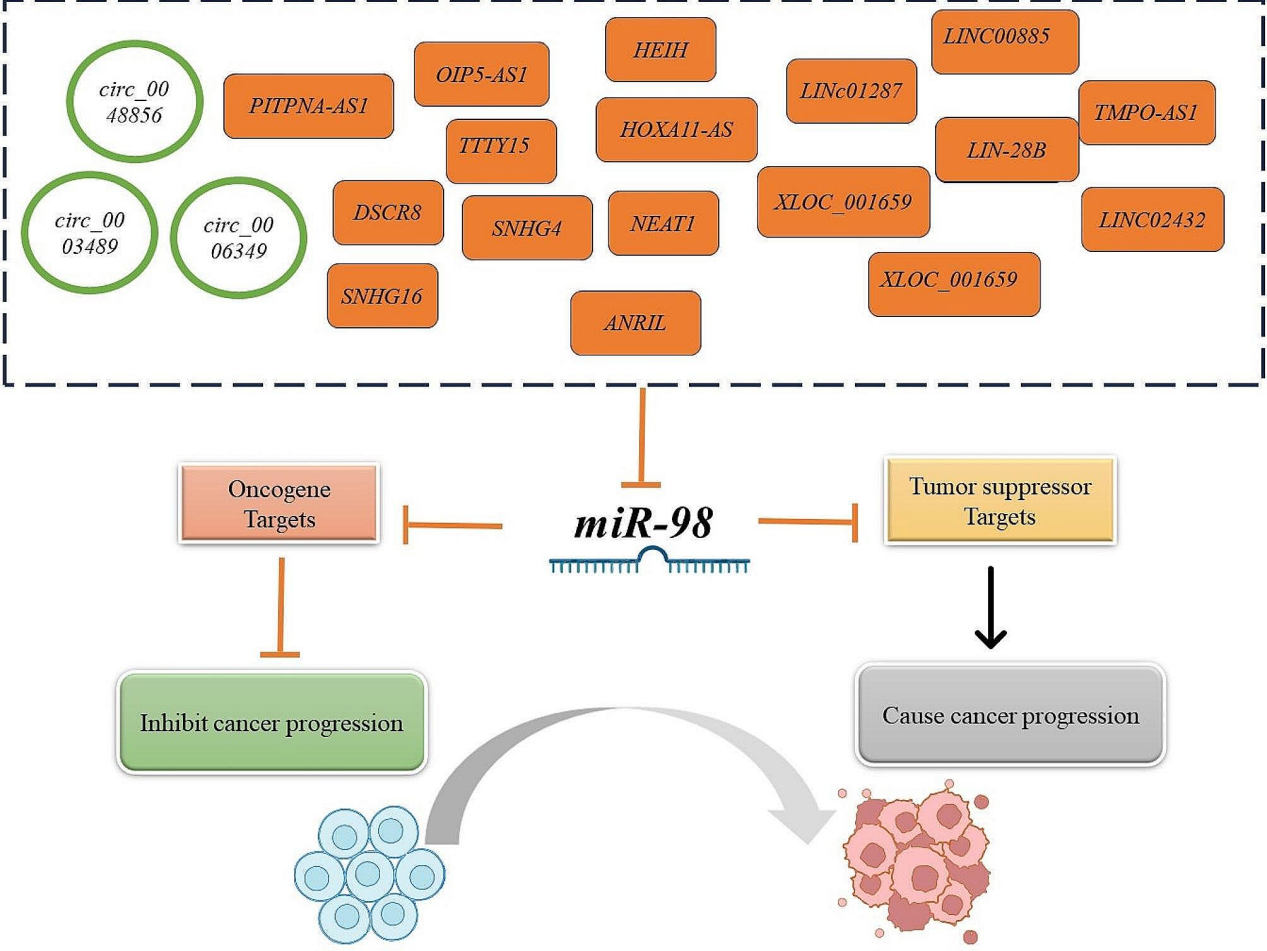

## Introduction

Cancer remains a significant global health issue due to its increasing cases and high mortality [1]. Despite progress in cancer research and improved detection and treatment methods, it continues to cause major societal and economic burdens [2]. Research is now focused on finding new biomarkers for early diagnosis and prognosis, as well as identifying potential therapeutic molecular targets. Non-coding RNAs (ncRNAs), including microRNA (miRNA), long non-coding RNA (lncRNA), and circular RNA (circRNA), have emerged as significant epigenetic factors in cancer development and progression [3–5].

MicroRNAs are small non-coding RNA molecules, typically 19–25 nucleotides in length, that regulate gene expression post-transcriptionally. These molecules play critical roles in numerous biological processes, including cell growth, differentiation, apoptosis, and metabolism [6]. In the context of oncology, miRNAs have garnered significant attention for their intricate roles in tumorigenesis, acting either as oncogenes or tumor suppressors [7]. Dysregulation of miRNAs can impact a wide range of cellular pathways, leading to uncontrolled proliferation, evasion from apoptosis, angiogenesis, and metastasis. Additionally, due to their stability in bodily fluids, miRNAs hold promise as diagnostic and prognostic biomarkers, providing non-invasive tools to monitor disease progression and therapeutic response [8].

Long noncoding RNAs, which are over 200 nucleotides in length and do not encode proteins, are important in regulating transcription and other cellular processes [9, 10]. Circular RNAs, characterized by their unique circular structure and stability, play a role in gene regulation as miRNA sponges and are linked to cancer progression [11]. Their dysregulation can contribute to cancer by acting as oncogenes or tumor suppressors [12].

Among the myriad of miRNAs studied in oncology, *miR-98* has risen to prominence owing to its intriguing roles in various human cancers. Initial studies reported differential expression of *miR-98* in tumor tissues compared to adjacent normal tissues, hinting at its potential relevance in carcinogenesis. Research unveiled *miR-98*’s capacity to modulate multiple signaling pathways, influencing tumor growth, metastasis, and therapy resistance.

Several studies have indicated that *miR-98* can act as both a tumor suppressor or an oncogene, depending on the cancer type and cellular context. Its multifaceted nature has led researchers to investigate its mechanistic roles and potential as a therapeutic target in greater depth [7].

This review aims to provide a comprehensive overview of the current knowledge surrounding *miR-98* in the context of human cancer. We will explore the molecular and cellular mechanisms by which *miR-98* contributes to cancer progression, its potential utility as a diagnostic and prognostic biomarker, and the emerging therapeutic strategies targeting this miRNA.

### Basics of microRNA-98

#### Biogenesis and molecular characteristics of miR-98

**Fig. 1:**
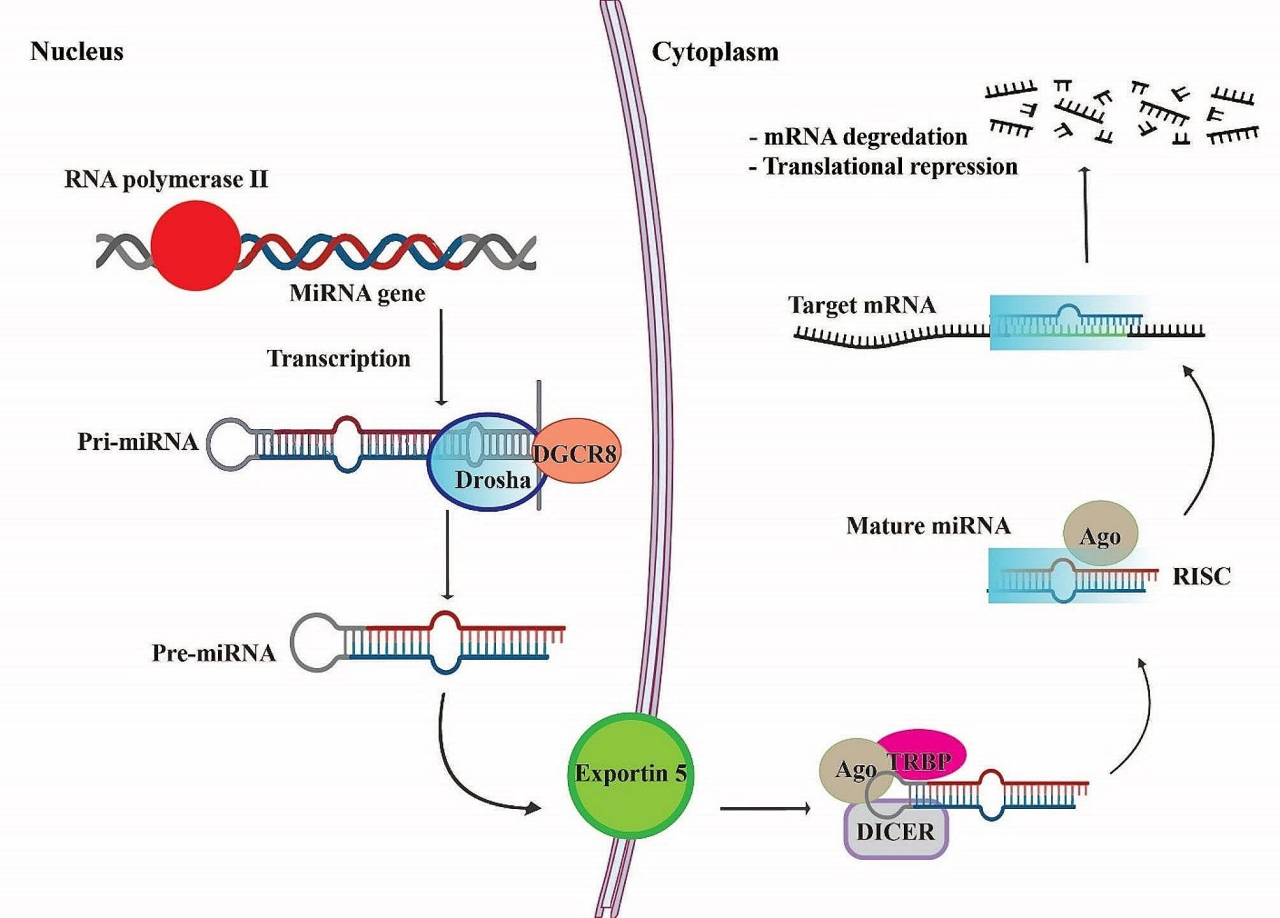
The process of miRNA biogenesis. Pre-miRNAs are created after RNAPII transcribes miRNA genes to pri-miRNAs, which are ultimately produced when Drosha cleaves pri-miRNAs. Pre-miRNAs are transferred to the nucleus and into the cytoplasm via Exportin5, where Dicer will turn them into mature miRNAs. The combination of mature miRNAs with AGO2 creates RISCs, which are essential for regulating gene expression

*MiR-98* is an intronic miRNA found on chromosome X (Xp11.22) and one of the twelve members of the *let-7* miRNA family [13]. *MiR-98* is initially transcribed as primary miRNAs (pri-miRNAs) in the nucleus by RNA polymerase II. Primary *miR-98* undergoes processing by the Drosha-DGCR8 complex, resulting in a precursor hairpin structure termed pre-*miR-98* [14]. This precursor is then exported to the cytoplasm via Exportin-5, where it is further cleaved by the enzyme Dicer to generate the mature *miR-98* molecule (Fig. 1) [15, 16].

The length of mature *miR-98-5p* and *miR-98-3p* is 22 nucleotides [17, 18]. Its specific sequence and secondary structure contribute to its target recognition and binding properties. Notably, the “seed sequence” of *miR-98*, typically spanning nucleotides 2–8 from its 5’ end, plays a critical role in target mRNA recognition and binding [19].

**Fig. 2:**
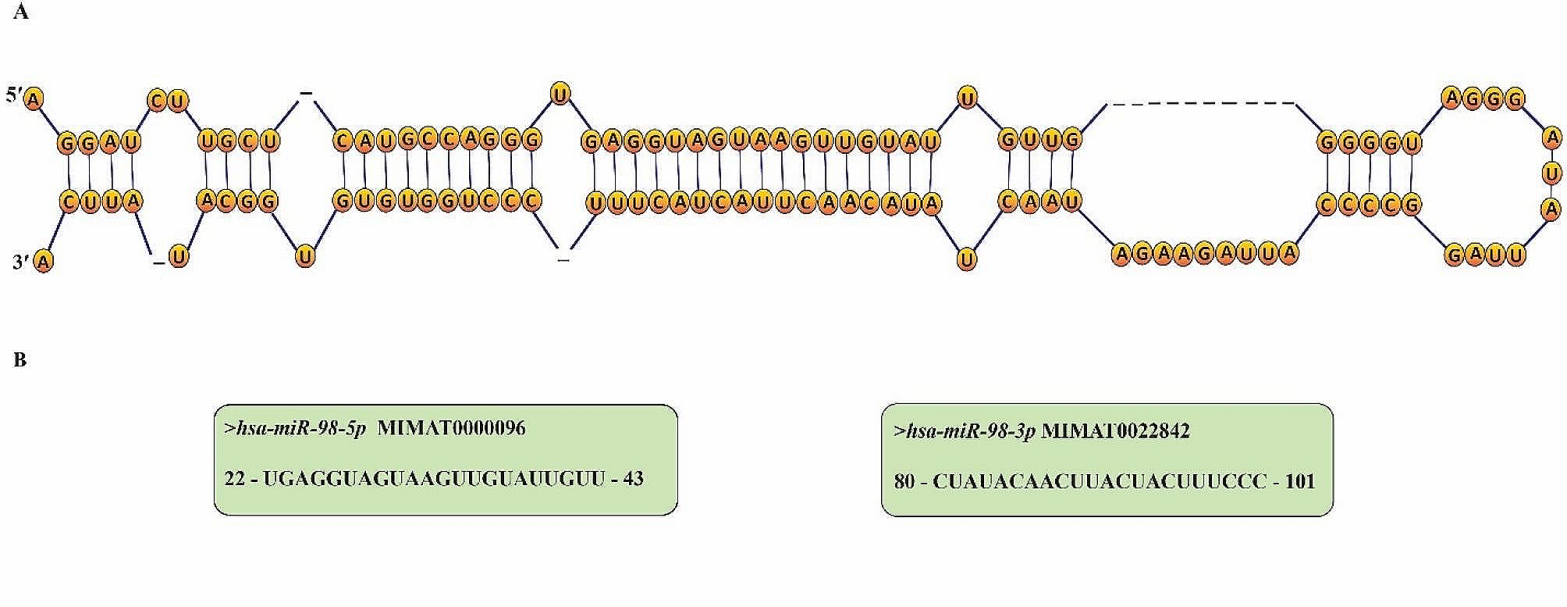
(**A**) *miR-98* family sequence structure. (**B**) It has two mature sequences, hsa-*miR-98-5p* (MIMAT0000096, *miR-98-5p*) and hsa-*miR-98-3p* (MIMAT0022842, *miR-98*-*3p*)

Mature miRNAs *miR-98-5p* and *miR-98-3p* are produced from the opposite arms of the stem-loop of pre-*miR-98* (Fig. 2A) [20]. The stability and functionality of these miRNAs vary in their biological characteristics. The “guide strand” *miR-98-5p* and the “passenger” strand *miR-98-3p* are produced by the *miR-98* hairpin, as shown in Fig. 2B. Deep sequencing data indicates that *miR-98-5p* is more common than *miR-98-3p* [18, 21].

The biogenesis of *miR-98* can be influenced by various cellular factors and conditions. For example, mutations or alterations in components of the Drosha or Dicer complexes can impact *miR-98* maturation [22, 23]. Additionally, external factors like cellular stress or specific signaling pathways can modulate the expression and maturation of *miR-98*, highlighting the intricate regulatory network governing its biogenesis [24].

#### Physiological roles of miR-98 in cellular functions

Under normal physiological conditions, *miR-98* often plays a role in regulating cellular growth. By targeting specific mRNAs involved in cell cycle progression, *miR-98* can fine-tune the balance between proliferation and quiescence. *MiR-98* has been implicated in cellular differentiation processes in various tissues [25]. For instance, in neuronal development, *miR-98* may modulate the differentiation of neural progenitors into mature neurons by regulating key transcription factors or signaling molecules [26]. Additionally, the balance between cell survival and programmed cell death is crucial for tissue homeostasis. *MiR-98* can influence this balance by targeting mRNAs associated with apoptosis, either promoting or inhibiting the process depending on the cellular context [27].

Furthermore, cells often encounter various forms of stress, such as oxidative stress, nutrient deprivation, or DNA damage. *MiR-98* contributes to the cellular stress response by modulating the expression of stress-responsive genes, aiding in either cellular adaptation or the initiation of cell death pathways [28]. Emerging evidence suggests that *miR-98*, like other miRNAs, can be packaged into extracellular vesicles, facilitating intercellular communication. Through this mechanism, *miR-98* may influence neighboring or distant cells, impacting tissue function and homeostasis [29].

#### Functional roles of miR-98 in cancer progression

**Fig. 3:**
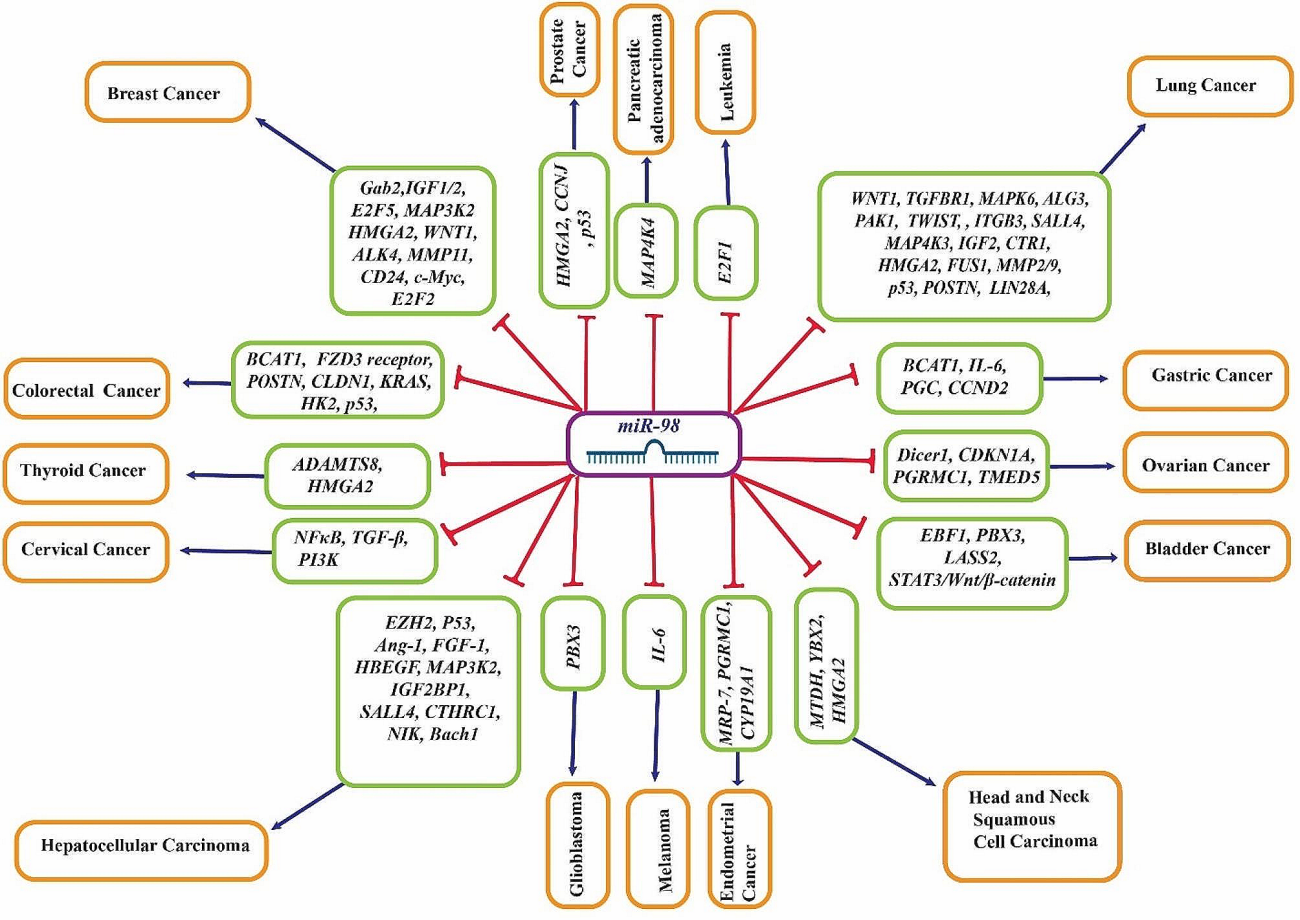
Comprehensive illustration of the interactions between *miR-98* and its main target genes

*MiR-98* plays a multifaceted role in cancer progression, exhibiting both oncogenic and tumor-suppressive properties, depending on the cancer context and microenvironment. Its dual role is exemplified by studies showing its contrasting functions in different cancer types (Fig. 3) (Table 1). For instance, while *miR-98* suppresses tumor growth in lung cancer, it promotes breast cancer progression [30, 31].

**Table 1:** The potential targets of *miR-98* and their functions in different cancers

Cancer Types	Target Gene	Tumor Suppressor or Oncogene	Function in Cancer	Ref.
Breast Cancer	*Gab2*	Tumor Suppressor	Proliferation, migration, and invasion	[32]
	*IGF1*	Tumor Suppressor	Cancer cell progression	[33]
	*IGF2*	Tumor Suppressor	Re-sensitizing herceptin-resistant cells	[34]
	*E2F5*	Tumor Suppressor	Development, proliferation, and migration	[35]
	*MAP3K2*	Tumor Suppressor	Cancer progressions and prognosis	[36]
	*HMGA2*	Tumor Suppressor	proliferation, invasion, migration; and apoptosis	[37]
	*WNT1*	Tumor Suppressor	Inhibition of cancer cells	[38]
	*ALK4*	Tumor Suppressor	Tumor growth, invasion, and angiogenesis	[39]
	*MMP11*			
	*CD24*	Unknown	Cancer cell regulation	[40]
	*c-Myc*	Tumor Suppressor	E2 response pathway	[41]
	*E2F2*			
Lung Cancer	*WNT1*	Tumor Suppressor	Cell viability and malignant colony formation	[38]
	*TGFBR1*	Tumor Suppressor	Cell proliferation, migration, invasion, and apoptosis	[42, 43]
	*MAPK6*	Tumor Suppressor	Cancer progression	[44]
	*ALG3*	Tumor Suppressor	Cell proliferation, migration, invasion, and apoptosis	[45]
	*PAK1*	Tumor Suppressor	Cell proliferation, migration, invasion, and apoptosis	[19]
	*TWIST*	Tumor Suppressor	Migration and invasion	[46]
	*ITGB3*	Tumor Suppressor	Cisplatin resistance, malignancy, and glycolysis	[47]
	*MKP1*	Tumor Suppressor	Glycolysis, proliferation, and apoptosis	[48]
	*SALL4*	Tumor Suppressor	Proliferation, migration, and invasion	[49]
	*MAP4K3*	Tumor Suppressor	Proliferation and apoptosis	[50]
	*IGF2*	Tumor Suppressor	Paclitaxel resistance	[36]
	*CTR1*	Tumor Suppressor	Cisplatin sensitivity	[51]
	*HMGA2*	Tumor Suppressor	Cisplatin sensitivity, EMT, and metastasis	[52, 53]
	*FUS1*	Tumor Suppressor	Regulatory network	[54]
	*MMP2/9*	Tumor Suppressor	Migration and invasion	[55]
	*p53*	Oncogene	Drug efficacy	[56]
	*POSTN*	Tumor Suppressor	EMT	[52]
	*LIN28A*	Tumor Suppressor	Cancer metastasis	[55]
Colorectal Cancer	*BCAT1*	Tumor Suppressor	Cancer progression	[57]
	*FZD3 receptor*	Tumor Suppressor	Tumor proliferation and metastasis	[58]
	*POSTN*	Tumor Suppressor	Apoptosis and EMT	[59]
	*CLDN1*	Tumor Suppressor	Cell proliferation, migration, and invasion	[60]
	*KRAS*	Not Specified	-	[61]
	*HK2*	Tumor Suppressor	Warburg effect, glycolysis, and in cancer cells proliferation	[61]
	*P53*	Not Specified	-	[62]
Hepatocellular Carcinoma	*EZH2*	Tumor Suppressor	Cell proliferation	[63]
	*P53*	Tumor Suppressor	Apoptosis	[64]
	*Ang-1*	Tumor Suppressor	Angiogenesis	[64]
	*FGF-1*			
	*HBEGF*	Tumor Suppressor	Clinical prognosis	[65]
	*MAP3K2*	Tumor Suppressor	Cell proliferation	[66]
	*IGF2BP1*	Tumor Suppressor	Cell proliferation and apoptosis	[67]
	*CTHRC1*	Tumor Suppressor	Cell proliferation, migration, and invasion	[18]
	*SALL4*	Tumor Suppressor	Cell proliferation, migration, invasion and EMT	[68]
	*NIK*	Tumor Suppressor	Cell proliferation, migration, and invasion	[69]
	*Bach1*	Tumor Suppressor	Cancer progression	[70]
Prostate Cancer	*HMGA2*	Tumor suppressor	Cancer development and progression	[71]
	*CCNJ*	Tumor suppressor	Cancer growth	[72]
	*p53*	-	-	[73]
Pancreatic adenocarcinoma	*MAP4K4*	Tumor Suppressor	Cell proliferation and migration	[74]
Gastric Cancer	*BCAT1*	Tumor Suppressor	Chemoresistance	[75]
	*IL-6*	Tumor Suppressor	-	[76]
	*PGC*	Not Specified	Cancer development	[77]
	*CCND2*	Tumor Suppressor	Cancer proliferation and apoptosis	[78]
Ovarian Cancer	*Dicer1*	Oncogene	Cisplatin resistance	[79]
	*CDKN1A*	Oncogene	Cisplatin resistance	[80]
	*PGRMC1*	Tumor Suppressor	Cancer proliferation and chemotherapy resistance	[81]
	*TMED5*	Tumor Suppressor	Reducing malignancy	[82]
Bladder Cancer	*EBF1*	Tumor Suppressor	Cancer development	[83]
	*PBX3*	Tumor Suppressor	Cell proliferation and apoptosis	[84]
	*LASS2*	Oncogene	Proliferation, drug resistance, and apoptosis	[85]
	*STAT3*	Tumor Suppressor	Cell proliferation, apoptosis, migration, and invasion	[86]
	*Wnt*			
	*β-catenin*			
Leukemia	*E2F1*	Tumor Suppressor	Cell proliferation and chemosensitivity	[87]
Thyroid Cancer	*ADAMTS8*	Tumor Suppressor	Cell progression	[88]
	*HMGA2*	Tumor Suppressor	Cell growth and apoptosis	[89]
Cervical Cancer	*PI3K*	Tumor Suppressor	Cancer progression	[90]
	*NFκB*	Tumor Suppressor	Cell proliferation and anti-apoptosis	[91]
	*TGF-β*			
Head and Neck Squamous Cell Carcinoma	*MTDH*	Tumor Suppressor	Cell proliferation, migration, and invasion	[92]
	*YBX2*	Tumor suppressor	Cancer Progression	[93]
	*HMGA2*	Tumor Suppressor	EMT and metastasis	[42]
Endometrial Cancer	*MRP-7*	Tumor suppressor	Drug resistance	[94]
	*PGRMC1*	Tumor Suppressor	Cell proliferation	[95]
	*CYP19A1*			
Melanoma	*IL-6*	Tumor Suppressor	Cancer metastasis	[96]
Glioblastoma	*PBX3*	Tumor Suppressor	Cell invasion and migration	[97]

*MiR-98* has been identified as having a dual nature in tumorigenesis. Specifically, in certain cancer scenarios, it promotes tumorigenesis, as evidenced by researchers’ findings in gastric cancer, where overexpression of *miR-98* led to increased cell growth and unfavorable patient outcomes [75]. This oncogenic potential might be driven by its ability to target and suppress tumor suppressor genes or pathways. Conversely, *miR-98* has also been reported to function as a tumor suppressor, such as in hepatocellular carcinoma, where it inhibits oncogenic pathways or directly targets genes that drive tumorigenesis [69].

**Fig. 4:**
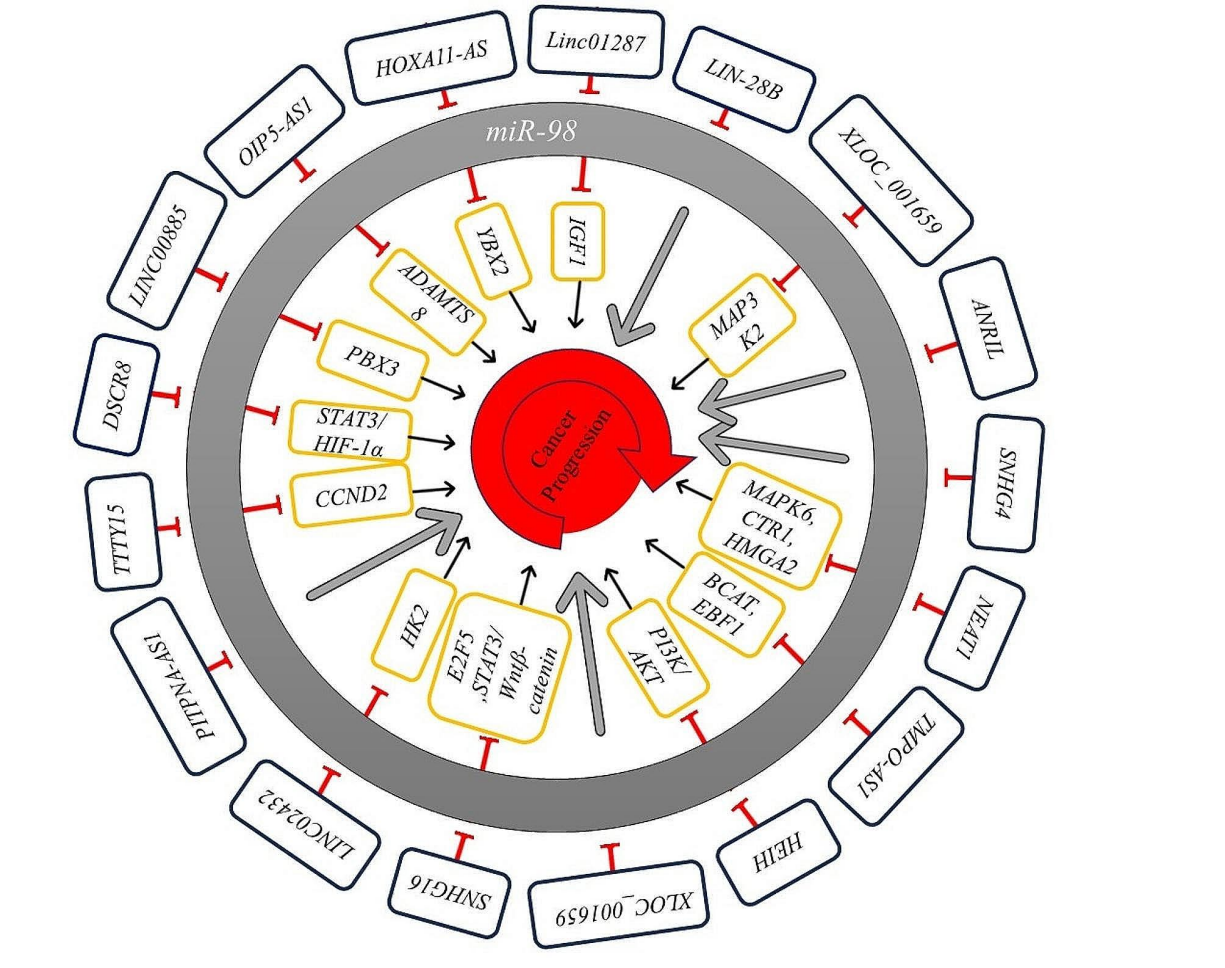
A detailed overview of LncRNAs’ interactions with *miR-98* in human malignancies

A variety of epigenetic elements are known to impact *miR-98*. Notably, current research is largely centered on how non-coding RNAs, such as lncRNA (long non-coding RNA) and circRNA (circular RNA), interact with *miR-98* (Fig. 4).

*MiR-98* has profound effects on cancer cell proliferation, apoptosis, and cell cycle regulation, according to research into its role in cellular mechanisms. It can modulate gene expression essential for cell growth, as exemplified by its ability to reduce cell proliferation in colorectal cancer cells, potentially via the *IGF1R* signaling pathway [98]. Apoptosis, a key mechanism in cancer control, is also influenced by *miR-98*. In glioma cells, *miR-98* has been found to induce apoptosis by targeting the *HMGA2* gene [99]. Moreover, its interaction with the cell cycle machinery can determine cell fate, with studies in cervical cancer revealing that its overexpression can induce G1 cell cycle arrest [91].

Furthermore, *miR-98* plays a significant role in metastasis and angiogenesis, essential processes in cancer spread and growth. Through the regulation of EMT, *miR-98* suppresses EMT and metastasis, as observed in bladder cancer, where it targets the IL-6/STAT3 signaling pathway [100]. Moreover, it can inhibit metastatic dissemination by regulating the degradation of the extracellular matrix, as evident in osteosarcoma, where *miR-98* curbs metastasis by targeting *MMP2* [101]. In pancreatic cancer, *miR-98* has been shown to attenuate angiogenesis by directly targeting *VEGFA*, underscoring its role in vascular dynamics within tumors [102].

In conclusion, *miR-98* emerges as a fundamental player in the complex landscape of cancer progression, dealing with tumorigenesis, cellular mechanisms, metastasis, and. Its role, whether tumor-promoting or tumor-suppressing, is highly dependent on the cancer type and microenvironment, emphasizing the necessity for context-specific therapeutic approaches targeting *miR-98* in cancer treatment.

### Patterns of miR-98 expression in different cancer types

#### Breast Cancer (BC)

BC is the most common cancer in women worldwide [103, 104]. At this point, targeted therapy, radiation therapy, chemotherapy, endocrine therapy, and surgical removal are the main methods of treatment [105]. Although the prognosis for breast tumors is improving, the condition is still the primary cause of mortality from cancer in women [103].

In breast cancer tissues and cell lines, *miR-98* expression has been frequently reported to be downregulated. This reduced expression is often associated with more aggressive tumor subtypes and poorer patient prognoses. Notably, the level of *miR-98* has been inversely correlated with metastatic potential in several breast cancer studies.

Researchers demonstrated the down-regulation of *miR-98-5p* in tumor tissues and MCF-7 breast cancer cells. Concurrently, they observed an up-regulation of *Gab2*, which countered by the transfection with *miR-98*-5p, led to significant inhibition of proliferation, migration, and invasion of MCF-7 cells [32]. Furthermore, other investigators elaborated on the oncogenic nature of *Linc01287* that sponge *miR-98-5p* and the negative regulation of *IGF1* by *miR-98-5p*, respectively. Overexpression of *miR-98* or knockdown of *Linc01287* resulted in an inhibitory effect on breast cancer cell progression, highlighting a potential therapeutic pathway in breast cancer treatment [33, 106].

The long noncoding RNA *SNHG16* promoted breast cancer cell migration by acting as a competitive endogenous RNA (ceRNA) for *E2F5* by binding with *miR-98*, while the *miR-98*-*5p/IGF2* axis affected herceptin sensitivity in HER2 positive breast cancer. Specifically, upregulation of *miR-98-5p* led to decreased *IGF2* expression, hence re-sensitizing herceptin-resistant cells [34, 35].

In a different perspective, experts revealed that submicron silica particles (SM-SiO2s) suppressed growth in various cancer cells, including breast cancer, by regulating the *XLOC_001659*/*miR-98*-*5p/MAP3K2* pathway, suggesting a broader spectrum of *miR-98*-*5p*’s anti-cancer effects [66]. Additionally, it was found that *miR-98* hindered proliferation, invasion, and migration while promoting apoptosis in breast cancer cells by targeting *HMGA2*, emphasizing the significance of *miR-98* in controlling multiple facets of cancer progression [37].

Moreover, exploratory studies broadened the scope to lung cancer and the effects of newly synthesized heterosteroids on miRNA expressions in MCF-7 breast cancer cells respectively. It was found that aspirin treatment induced the expression of *miR-98*, depressing *WNT1* in lung cancer cells [38]. It was noted that while tamoxifen up-regulated *miR-98* expression, new heterosteroids significantly down-regulated it, suggesting a potential for reducing drug resistance [42].

Comprehensive analyses supported *miR-98*’s important role in regulating tumor growth, invasion, and angiogenesis by down-regulating *ALK4* and *MMP11*, as well as its potential predictive value as a biomarker in breast cancer patients [39, 43]. A specific study explored the potential of *miR-98* in predicting Sentinel Lymph Node Metastasis in ER+/HER2-breast cancer, developing a model that showed a significant association between *miR-98* and SLNM, however, the direction of *miR-98* regulation wasn’t detailed [106]. Additionally, it was demonstrated that dihydromyricetin could potentiate the efficacy of Herceptin in SKBR3 cells by up-regulating *miR-98-5p*, hence inhibiting IGF1R/HER2 dimer formation and consequently reversing Herceptin resistance [107].

*MiR-98* was discovered to be differentially expressed in the HER2 + subtype across different breast cancer subtypes [108]. Employing algorithms, a regulatory interaction between *miR-98* and the *CD24* gene in breast cancer was identified, implying that *miR-98* may play a functional role via *CD24* targeting [40]. Deep sequencing also revealed a decrease in *miR-98/Let-7* family miRNA expression in breast tumors compared to normal tissues, which aligns with the transition from noninvasive to invasive carcinomas [109].

The interaction between *miR-98* and drug response was discovered, revealing that *miR-98*, along with other miRNAs, could affect docetaxel sensitivity [110]. Furthermore, estradiol (E2) induced the upregulation of *miR-98* and other miRNAs in MCF-7 breast cancer cells, which resulted in a decrease in c-Myc and E2F2 protein levels, demonstrating *miR-98*’s role in the E2 response pathway in breast cancer scenarios [41]. The potential association of *miR-98* with breast cancer was identified using network-based algorithms [111].

Downregulation of *miR-98* in breast cancer biology has been linked to aggressive tumor subtypes and adverse patient outcomes. *MiR-98* modulation could be beneficial in the treatment of breast cancer by influencing tumor growth, invasion, and drug responses. In addition to breast cancer, *miR-98* could possibly be useful in the diagnosis, prognosis, and treatment of other cancers.

#### Lung cancer

Lung cancer is one of the most common cancers globally and contributes significantly to cancer-related death [112]. Based on their histological features, small-cell lung cancer (SCLC) and non-small-cell lung cancer (NSCLC) are the two main subtypes of lung carcinoma. The cause of about 85% of lung cancers is NSCLC [113]. Contrastingly, in certain subtypes of lung cancer, particularly non-small cell lung carcinoma (NSCLC), *miR-98* has shown elevated expression. This upregulation has been linked to increased tumor growth and resistance to certain therapeutic agents, suggesting an oncogenic role in this context.

A specific study found that aspirin could improve lung cancer by targeting the *miR-98*/*WNT1* axis. This targeted intervention reduced cell viability and formed malignant colonies. In lung cancer cells, aspirin activated *miR-98*, which decreased *WNT1* expression. This discovery illuminates aspirin’s lung cancer treatment mechanism [38]. Similar studies have shown that *miR-98* regulates several molecular interactions, including *miR-98*-*5p/TGFBR1*, *ALG3*, and *PAK1*. These regulatory interactions are crucial to lung cancer cell proliferation, migration, invasion, and apoptosis [45].

Several studies have found *miR-98*-related molecular networks, including the *NEAT1*/*hsa-miR-98-5p/MAPK6* axis and the *XLOC_001659*/*MAP3K2* pathway. The studies show that *miR-98* affects lung cancer progression in a variety of cancer environments [44, 66]. Scientific studies on circular RNAs like *circ_0048856* and *circ_0006349*, as well as interactions between integrin β3 (*ITGB3*) and *miR-98*, reveal challenging regulatory mechanisms. These interactions regulate lung cancer pathogenesis, including cisplatin resistance, malignancy, glycolysis, and in vivo tumor formation via targeting *ITBG3* and *MKP1* [47, 48, 54].

Researchers found a significant inverse relationship between lncRNA *ANRIL* and *miR-98* in lung cancer cells, demonstrating that suppressing *ANRIL* increases *miR-98* expression, preventing cisplatin resistance [114]. Another study found that lncRNA *SNHG4* regulates *miR-98-5p*, affecting lung cancer cell proliferation, migration, and invasiveness [115].

Researchers also noted that *miR-98* inhibits *TWIST* expression, which inhibits NSCLC cell migration and invasion, making it a potential tumor suppressor [46]. Jiang et al. and Ni et al. found that *miR-98* modulates *TGFBR1* and *ITGB3* to inhibit cancer cell proliferation, migration, and invasion [48, 49]. Another study found that increasing *miR-98* expression could hinder NSCLC progression by inhibiting the SALL4 protein [50].

Another example of complexity was uncovered in a study that identified a regulatory network involving miR-93, *miR-98*, and *miR-197*. These microRNAs impact the expression of the tumor suppressor gene *FUS1*, and an observed overexpression of *miR-93* and *miR-98* in small-cell lung cancer was documented [55]. Researchers found that curcumin suppressed lung cancer metastasis by increasing *miR-98* expression, which downregulated *LIN28A, MMP2*, and *MMP9* [55]. In another study, thiostrepton, an anti-cancer stem cell agent, increased tumor suppressor *miR-98* levels to inhibit NSCLC cell growth and improve chemotherapy efficacy when combined with gemcitabine [52].

Researchers found that *miR-98-3p* was the least expressed dysregulated exosomal miRNA in lung adenocarcinoma (LUAD) patients. Although exosomal miR-7977 was the main focus as a novel biomarker for LUAD, LUAD patients had lower serum *miR-98-3p* than controls [116].

The researchers discovered that using a mimic to upregulate *miR-98-5p* reduced cell proliferation and increased apoptosis in NSCLC cells by targeting *MAP4K3*, indicating a potential pathway for suppressing NSCLC progression [50]. Another study found that the circular RNA *hsa_circ_0003489* affects the resistance to paclitaxel in NSCLC through the *miR-98*-*5p/IGF2* axis. Inhibiting *hsa_circ_0003489* decreased IGF2 expression by sponging *miR-98-5p* and improved paclitaxel sensitivity in resistant NSCLC cells [36]. Lastly, in lung adenocarcinoma, decreased hsa-*miR-98* expression in induced pulmosphere cells may indicate a cancer stem cell phenotype [56].

hsa-*miR-98-5p* suppresses *CTR1* gene expression, affecting NSCLC cell cisplatin sensitivity. Moreover, lncRNA *NEAT1* sponging hsa-*miR-98-5p* increased EGCG-induced *CTR1* expression and cisplatin sensitivity. This suggests a lung cancer treatment strategy to overcome cisplatin resistance [53]. *miR-98* overexpression inhibits epithelial-to-mesenchymal transition (EMT) and metastasis by targeting *HMGA2*, which represses *POSTN*, suggesting a therapeutic *miR-98*-*HMGA2-POSTN* pathway [53].

hsa-*miR-98-5p* expression levels in NSCLC patients were associated with a higher objective response rate (ORR) to radiotherapy, suggesting it may be a biomarker [117].

In NSCLC A549 cells, modulating hsa-*miR-98-5p* with (-)-epigallocatechin-3-gallate (*EGCG*) and cisplatin increased p53 and cisplatin-induced apoptosis, reducing tumor size and improving drug efficacy [57]. Also, it was found that *miR-98* upregulation increased HMGA2 protein levels and increased cisplatin sensitivity in cisplatin-resistant human lung adenocarcinoma cells with a decrease in *miR-98* expression [52].

The findings showed notable differences in *miR-98* expression between benign pleural effusion (BPE) and lung adenocarcinoma-associated malignant pleural effusion (LA-MPE) samples when properly normalized [118]. Another study suggested using *miR-98* and *miR-205* expression levels to distinguish normal from cancerous lung squamous cell carcinoma samples [57]. Hsa-*miR-98*, along with other miRNAs in the *hsa-let-7* family, was expressed more in adenocarcinoma (AD) than squamous cell carcinoma (SCC) in transthoracic needle aspiration samples of NSCLC, emphasizing its role in classifying the cancer [58].

*miR-98* plays a multifaceted role in lung cancer, acting both as a potential oncogene and tumor suppressor. Numerous studies have revealed its involvement in numerous molecular interactions and pathways that influence lung cancer progression and treatment resistance. Because of *MiR-98*’s multiple roles, its regulatory mechanisms are critical for lung cancer treatment and biomarker development.

#### Colorectal cancer (CRC)

Colorectal cancer (CRC) is the third most common type of cancer diagnosed and the second most common cause of cancer-related deaths [59]. *MiR-98* expression patterns in the CRC are diverse. While some studies have reported its downregulation, associating it with advanced stages and metastasis, others have found it to be upregulated, especially in early-stage tumors, indicating its potential role in tumor initiation.

Serum *miR-98* downregulation is associated with aggressive clinical characteristics and decreased survival in CRC patients, making it a potential prognostic indicator for overall and disease-free survival [60]. This essential understanding lays the foundation for *miR-98* regulatory dynamics research in the CRC.

A study found that the long non-coding RNA *TMPO-AS1* sponges *miR-98-5p* to upregulate BCAT1, boosting CRC progression [61]. In a computational investigation, *miR-98-5p* was found to target *FZD3* in the Wnt signaling pathway to decrease CRC tumor proliferation and metastasis. The direct binding of *miR-98-5p* to the *FZD3* mRNA 3’UTR suppressed colorectal cancers [119]. These connections show *miR-98*’s integrative role in molecular regulatory networks.

Overexpression of *miR-98* inhibited the up-regulation of periostin (*Postn*), which facilitates colon cancer cells in apoptosis and transition from epithelial to mesenchymal [62]. *Postn* is a *let-7a/miR-98* target gene. It has also been demonstrated that boosting *miR-98* in CRC cells decreased cell proliferation, migration, and invasion while increasing cell death through decreasing *CLDN1* [120].

Understanding CRC molecular features may also require *miR-98* expression. Significant downregulation of *miR-98-5p* in *KRAS* mutant CRC tissues compared to wild-type was observed, suggesting a molecular signature that could enhance CRC molecular understanding and aid in identifying novel biomarkers [62].

Interestingly, *miR-98* directly targeted hexokinase 2 (*HK2*) to suppress the Warburg effect and reduce glycolysis and proliferation in colon cancer cells [63]. The metabolic regulation of colon cancer cells by *miR-98* offers new therapeutic possibilities.

According to P53 status, radiation and SN38 treatments in colon cancer cells modulated miRNA, cytokine, and chemokine expression. *miR-98* was highly elevated and associated with colon cancer pathways and cytokine or chemokine expression. This suggested that *miR-98* may treat colon cancer, especially in a P53-dependent way [121].

Several miRNAs changed between low- and high-grade intraepithelial dysplastic polyps in a porcine model of familial adenomatous polyposis. *miR-98* levels were significantly higher in high-grade polyps, implying a role in colon polyp premalignancy [122]. A study discovered that patients with polyps or adenomas exhibited higher *miR-98* expression than controls [123].

Serum levels of *miR-92a-3p* and *miR-98-5p* improved chemotherapy response prediction, although not enough to alter therapeutic decisions [63]. It was shown that *miR-98*-*5p*, along with clinical and pathological factors, can predict treatment results in metastatic CRC patients receiving first-line systemic therapy [64].

Moreover, in extracellular vesicle (EV) dynamics, investigators unraveled miRNA profiles within three distinct EV subtypes released from the human LIM1863 colon cancer cell line. In shed microvesicles (sMVs), *miR-98-5p* was selectively represented, depicting a selective enrichment of *miR-98-5p* in specific EV subtypes [65].

*miR-98* plays a multifaceted role in colorectal cancer (CRC) progression and prognosis, with varying expression patterns across different tumor stages and cellular functions. Its involvement spans molecular regulatory networks, affecting CRC cell proliferation, metastasis, apoptosis, and metabolic processes, with potential prognostic and therapeutic implications. Further exploration into *miR-98*’s intricate interactions and regulatory dynamics in CRC can provide valuable insights for diagnosis, prognosis, and targeted treatments.

#### Hepatocellular carcinoma (HCC)

Hepatocellular carcinoma ranks as the sixth most common and the fourth deadliest cancer worldwide, predominantly affecting liver cells [124]. In HCC, *miR-98* often exhibits decreased expression, and its downregulation has been connected to increased cell proliferation, invasion, and worse overall survival rates in patients.

A critical analysis of *miR-98*’s function in HCC found that HCC tissues and cell lines had significantly lower *miR-98* expression than nearby non-tumorous tissues and the hepatic cell line LO2. It was found that *miR-98* decreased HCC cell proliferation by targeting *EZH2* and suppressing the Wnt/β-catenin signaling pathway, resulting in a G0/G1 cell cycle arrest [36].

The overexpression of *P53* and downregulation of *Ang-1* and *FGF-1* genes in HepG2 cells showed *miR-98*’s pro-apoptotic and anti-angiogenic effects [67]. Researchers found that *miR-98-5p* expression levels in HCC patients’ serum decreased significantly, showing its potential to target and down-regulate the *HBEGF* gene, affecting disease prognosis when analyzed alongside MRI data [65].

In terms of drug resistance, the lncRNA *HEIH* regulates *miR-98-5p* in the PI3K/AKT signaling pathway to mediate Sorafenib resistance in HCC. In HCC cells, suppression of *miR-98-5p* activates the PI3K/AKT pathway, boosting Sorafenib resistance [125]. Researchers also looked at *XLOC_001659*/*miR-98-5p*/*MAP3K2*, a novel molecular axis that regulates HCC proliferation via submicron silica particles.

*MiR-98-5p* was significantly downregulated in HCC tissues and cell lines. By targeting *IGF2BP1*, overexpression of this miR reduced HCC cell growth and promoted apoptosis [68]. *MiR-98*’s role in macrophage polarization has also been studied, with a focus on its impact on TAM-driven HCC development and invasion [126, 127]. Further studies revealed that *miR-98* can downregulate genes such as *SALL4* and *CTHRC1*, reducing HCC cell malignancies and solidifying its function as a tumor suppressor [18, 70].

HBV-related HCC was connected to a distinct miRNA signature in a study on miRNAs. *MiR-98* was discovered in HBV-unrelated HCC and HBV infection, implying a greater role in disease pathogenesis. According to Gene Ontology (GO) and KEGG pathway analyses, several miRNAs, including *miR-98*, impact transcription regulation and MAPK signaling pathway-mediated protein phosphorylation, which is important in HCC and HBV infection [128].

*MiR-98-5p* downregulation in HBV-HCC tissues and cells increases apoptosis by decreasing NF-B-Inducing Kinase (*NIK*) expression [71]. *Lin-28B* overexpression inhibited let-7/*miR-98* family members, supporting *miR-98*’s tumor-suppressive action in HCC, implying that *miR-98* downregulation in HBV-HCC may limit tumor proliferation [129].

Researchers discovered that overexpression of *miR-98-5p* diminished *PPP1R15B* levels in HaCaT cells, enhancing apoptosis and decreasing cell proliferation. This finding sheds light on how inadequate levels of *miR-98-5p* in the circulatory system may contribute to diabetes-related skin hyperproliferation [130]. Furthermore, *miR-98-5p* downregulation was detected in ovarian cancer tissues associated with disease progression, implying that it may also inhibit tumor growth. These findings add to our understanding of the role of *miR-98* in cancer and other diseases, such as diabetes [70].

Scientific investigations have highlighted the potential of circulating miRNAs as markers of hepatocarcinogenesis progression in rats. Notably, elevated circulating *miR-98* levels were observed even at the early stages of hepatocarcinogenesis [131]. Different studies linked *miR-98* to clusters of circulating *let-7* family tumor suppressors in chronic hepatitis C patients, suggesting its potential in liver disease monitoring [132]. Another murine model showed *miR-98* dysregulation during liver neoplastic processes, suggesting it could be used as a biomarker for early HCC identification and HT1 damage development [71].

Research on the *let-7* microRNA family showed *miR-98* suppressed Bach1, a known HMOX1 gene repressor, to modulate *HMOX1* expression. In human hepatocytes, *miR-98* dramatically decreased Bach1 protein levels and increased *HMOX1* gene expression, suggesting a way to reduce oxidative injury [72].

In hepatocellular carcinoma (HCC), *miR-98* consistently exhibits reduced expression, which correlates with increased cell proliferation, invasion, and poorer patient survival rates. *MiR-98* has been linked to several key pathways in HCC, notably inhibiting the Wnt/β-catenin signaling pathway, targeting genes such as *EZH2* and *IGF2BP1*, and impacting drug resistance mechanisms. Moreover, the broad involvement of *miR-98* in various other cancers and diseases, such as diabetes and liver disease, underlines its potential as a therapeutic target and diagnostic marker across a spectrum of health conditions.

#### Prostate cancer (PC)

Prostate cancer is the second most frequent cancer in men and ranks sixth in terms of cancer-related mortality in men worldwide [133]. The expression of *miR-98* in prostate cancer (PCa) has been found to be context-dependent. Although certain studies indicate its downregulation, implying a tumor-suppressive role, others show its upregulation in castration-resistant subtypes, suggesting a role in therapeutic resistance.

A comprehensive analysis found significant upregulation of *miR-98* and related miRNAs like *miR-152-3p, miR-326*, and *miR-4289* in the plasma of PCa patients compared to healthy subjects, showcasing high diagnostic precision with an AUC of 0.88. Downregulation of these miRNAs was often linked with advanced cancer stages and unfavorable survival outcomes, suggesting potential diagnostic and prognostic relevance specific to PCa [134].

It was discovered that the long non-coding RNA, *NEAT1*, acts as a sponge for *miR-98*-5p, thus promoting the expression of the oncogene *HMGA2* in PCa. With *NEAT1* being notably up-regulated in PCa tissues and cell lines and its reduction halting the growth and invasion of certain cells, this sheds light on a crucial mechanism in PCa development and progression [73].

There’s evidence suggesting *miR-98* could be modulated by vitamin D administration. One study demonstrated that 1,25-VD transcriptionally induces *miR-98*, subsequently inhibiting specific cell growth and leading to the reduction of *CCNJ*, a particular mitotic control protein. This emphasizes the potential therapeutic role of vitamin D in PCa via *miR-98* modulation [72].

Supporting these findings, another research found a significant increase in levels of *miR-98-5p* and other miRNAs in the plasma of PCa patients, confirming the diagnostic potential. Further, specific miRNAs, such as *miR-152-3p*, have been associated with increased PCa cell proliferation and migration, underscoring their vital role in PCa progression [73].

A meta-analysis aimed at identifying co-deregulated miRNA genes highlighted a series of upregulated miRNAs, including *miR-98*, in recurrent PCa post-radical prostatectomy. These miRNAs were implicated in pivotal pathways regulating various cellular processes and modulating several significant proteins, illustrating a broad molecular interaction [135].

Another significant contribution was the development of a diagnostic model using plasma miRNAs. The study identified *miR-98-5p* and *miR-26b-5p* as potential molecular markers for distinguishing PCa cases, especially differentiating low-grade from high-grade prostate cancer [136].

Diving deeper into the intricate patterns of miRNA, a study explored the interaction between testosterone, dietary tomato carotenoids, and miRNA expression during the early phases of prostate carcinogenesis. It was concluded that diet can modulate specific miRNAs like *miR-98*, revealing new insights into dietary influences on prostate carcinogenesis [75].

Lastly, an investigation of six microRNAs, including *miR-98* revealed a decreased expression of *miR-98* in PCa patients compared to benign prostatic hyperplasia (BPH) cases. This reduced expression correlated with the Gleason grades of PCa, reinforcing the significance of *miR-98* as a valuable biomarker for PCa detection and prognosis [74].

In prostate cancer (PCa), *miR-98*’s expression is complex and varied, with research indicating both tumor-suppressive and resistance-associated roles. Significant studies have shown *miR-98*’s diagnostic precision in differentiating PCa patients from healthy subjects and its association with key pathways and oncogenes, such as the oncogene *HMGA2* via the lncRNA *NEAT1*. Additionally, the potential modulation of *miR-98* by factors like vitamin D and dietary tomato carotenoids emphasizes its multifaceted role in prostate cancer development, progression, and potential therapeutic interventions.

#### Pancreatic ductal adenocarcinoma (PDAC)

The incidence of PDAC, a highly aggressive disease that affects people all over the world, has increased recently [137]. The importance of *MiR-98* in pancreatic ductal adenocarcinoma has been highlighted by studies revealing its role in tumor progression and treatment resistance.

In a study on pancreatic adenocarcinoma (PAAD), a connection between glycolysis and the *LINC02432*/hsa-*miR-98-5p/HK2* molecular triad was identified. A higher hallmark glycolysis score, derived from the single-sample GSEA (ssGSEA) algorithm, was linked to a poorer prognosis for PAAD patients. An analysis from the TCGA and GEO databases revealed the *LINC02432*/hsa-*miR-98-5p/HK2* axis, which was found to be inversely related to ferroptosis. A higher ceRNA risk score was correlated with increased M0 macrophage infiltration in PAAD, associations with specific chemokines, the immune checkpoint gene SIGLEC15, and a positive relationship with the tumor mutation burden (TMB). Patients with elevated risk scores showed better responsiveness to drugs targeting *EGFR*, *MEK*, and *ERK* [138].

In another investigation, it was observed that *miR-98-5p* was significantly downregulated in PDAC tissues, influencing vital clinical outcomes. This downregulation affected PDAC cell behavior, promoting proliferation and migration. Through assays, *MAP4K4* was pinpointed as a direct target of *miR-98-5p*. The overexpression of *miR-98-5p* suppressed the MAPK/ERK signaling pathway, primarily by downregulating *MAP4K4*, suggesting its therapeutic potential in PDAC [32].

Although the downregulation of *miR-98* in PDAC suggests that it plays an important role in disease progression and has therapeutic potential, more research into *miR-98*’s role in PDAC is needed to fully understand its behavior.

#### Gastric cancer (GC)

Currently the second greatest cause of death globally, gastric cancer has emerged as one of the most common cancers [76]. *MiR-98* expression has been identified as a critical determinant in gastric adenocarcinomas, with altered levels correlating with tumor progression, metastasis, and treatment responses.

The overexpression of the long noncoding RNA *PITPNA-AS1* in GC correlates with poorer survival rates. This lncRNA directly interacts with *miR-98*-*5p*, inhibiting it and thereby leading to cisplatin treatment resistance in GC cells. Conversely, suppression of *PITPNA-AS1* curtailed cell growth and increased cisplatin sensitivity [77]. There’s also evidence that downregulation of *miR-98-5p* in CD44 + GC stem cells results in increased cancer cell stemness and chemoresistance by targeting BCAT1. However, *miR-98* overexpression could reverse such effects [77]. Additionally, *miR-98-5p* downregulation in GC tissues saw an upregulation in Treg/Th17-related factors. Overexpressing *miR-98-5p* can regulate this balance by targeting IL-6, with the balance being further modifiable by oleanolic acid through *miR-98-5p* upregulation, hinting at a treatment pathway for GC [78]. Furthermore, a ceRNA network linked to Pepsinogen C (PGC) expression in GC involving *miR-98-5p* was identified, showcasing a complex interplay between lncRNAs, circRNAs, and miRNAs in modulating PGC expression and influencing GC progression post-transcriptionally [79]. Another layer of complexity emerges with the interaction between lncRNA *TTTY15* and *miR-98*-5p. Silencing *TTTY15* slows GC progression by acting on *miR-98-5p* and downregulating cyclin D2 (*CCND2*) expression, as corroborated by increased *TTTY15* and *CCND2* expression, and decreased *miR-98-5p* in GC tissues and cell lines [78].

Studies into miRNA and mRNA signatures in hydroxycamptothecin (HCPT)-resistant GC cell lines identified 25 miRNAs, including *miR-98*, deregulated in HCPT-resistant cells, impacting cancer development, progression, and chemosensitivity [139]. Similarly, a noteworthy up-regulation of 22 out of 24 miRNAs, inclusive of *miR-98*, was found in GC compared to normal gastric tissue, suggesting a significant role in GC progression [79].

The intricate molecular interaction of *MiR-98* in gastric cancer indicates its potential as a diagnostic and therapeutic tool. Given its complex interactions and effects, there is an urgent need for comprehensive research to determine its precise role and potential applications in gastric cancer management.

#### Ovarian cancer (OC)

The seventh most common malignant tumor in women worldwide is ovarian cancer. About 239,000 cases of OC are reported annually, and 152,000 deaths have been attributed to the condition [80]. *MiR-98* has emerged as a significant factor in ovarian cancer, with its expression levels influencing various oncogenic pathways and patient outcomes.

It was found that *miR-98-5p* is enriched in cisplatin-resistant epithelial ovarian cancer (EOC) cells, promoting cisplatin resistance by hindering *miR-152* biogenesis through targeting Dicer1, which is correlated with poor outcomes in EOC patients [81]. Contrarily, it was shown that *miR-98-5p* is downregulated in ovarian cancer tissues, and its mitigative effects on cancer are hampered by overexpressed lncRNA *DSCR8*, which is known to promote cancer progression through a highlighted lncRNA *DSCR8*/*miR-98-5p*/*STAT3/HIF-1α* axis [70]. A mechanism was highlighted in which cancer-associated fibroblast-derived exosomal *miR-98-5p* promotes cisplatin resistance by downregulating *CDKN1A* [82]. Networks involving *miR-98*-5p, associating it with tumorigenesis, progression, and chemoresistance in ovarian serous cystadenocarcinoma and carboplatin-resistant ovarian cancer, were explored [81, 140]. It was demonstrated that *miR-98*, alongside *let-7*, targets and regulates *PGRMC1* expression, a component known to be tied to chemoresistance, suggesting a regulatory mechanism for *PGRMC1* expression in ovarian cancer [95]. Among a panel of miRNAs, *miR-98-5p* was identified as a biomarker for resistance to platinum-based chemotherapy in high-grade serous ovarian cancer (HGSC) [82].

On a broader scale, *miR-98* was recognized as significantly associated with survival in ovarian cancer patients, indicating a potential role in improving treatment decisions. While focusing on endometrial transition, aberrant expression of *miR-98* was associated with the transition into cancerous states, with *miR-98* being observed to have an inverse relationship with *PGRMC1* and *PGR* expression [97]. A contrasting scenario was revealed where the knockdown of *SNHG4*, known to reduce ovarian cancer cell malignancy, was reversed by *miR-98-5p* downregulation or *TMED5* overexpression [83].

The diverse roles of *miR-98-5p* in ovarian cancer, from influencing disease progression to chemoresistance, underscore its potential as a valuable biomarker. Given its diverse impacts, there is a pressing need for deeper research to optimize its application in therapeutic approaches for ovarian cancer.

#### Bladder cancer

Bladder cancer is a significant global health challenge and ranks among the top ten most common cancer types worldwide [84]. Bladder carcinoma research has consistently shown the critical role of *miR-98*, with its downregulation impacting tumor growth and response to chemotherapy.

Various molecular interactions involving *miR-98-5p* or *miR-98* that contribute to the progression and malignancy of bladder cancer through different axes and regulatory loops are explored. A feedback loop involving *TMPO-AS1*/*miR-98-5p/EBF1*, which significantly impacts the development of bladder cancer, was demonstrated; in this loop, *TMPO-AS1* is sponged by *miR-98-5p*, which subsequently targets *EBF1* [85]. An upregulation of *LINC00885* targeting the *miR-98-5p/PBX3* axis, promoting bladder cancer progression, was elucidated; here, an upregulation of *miR-98-5p* is observed to reduce cell proliferation and enhance cell apoptosis [86]. The upregulation of *miR-98* in bladder urothelial carcinoma tissues and cell lines, promoting proliferation and drug resistance while reducing apoptosis in bladder cancer cells, was highlighted; specifically, LASS2 is targeted by *miR-98*, and mitochondrial function is regulated, affecting chemoresistance [87]. Lastly, the upregulation of *SNHG16* in bladder cancer was revealed; it negatively regulates *miR-98* expression, which contributes to bladder cancer malignancy through the *miR-98*/*STAT3*/Wnt/β-catenin pathway axis. This showcases a complex regulatory interaction between *SNHG16*, *miR-98*, and *STAT3*, influencing the Wnt/β-catenin pathway and bladder cancer development [87].

The different interactions and regulatory loops involving *miR-98* in bladder cancer progression highlight its potential significance as both a therapeutic target and a diagnostic biomarker. Understanding these complex networks can offer promising avenues for enhancing bladder cancer treatment and diagnosis.

#### Leukemia

Leukemia involves the rapid growth of abnormal white blood cells, with types like acute myeloid leukemia (AML) and chronic lymphocytic leukemia (CLL) impacting adults differently [84]. In hematological malignancies like leukemia, the role of *miR-98* is intricate. For instance, in chronic lymphocytic leukemia, *miR-98* appears to be downregulated and is linked with disease progression. In contrast, in certain subtypes of acute myeloid leukemia, *miR-98* is found to be upregulated, potentially playing a role in the inhibition of cell differentiation.

Investigators provided insights into the role of *miR-98* and other microRNAs in different types of leukemia and their implications in treatment and prognosis. A significant reduction in the expression of *miR-32-5p, miR-98-5p*, and *miR-374b-5p* in Chronic Lymphocytic Leukemia (CLL) patients was observed, suggesting these miRNAs might act protectively against CLL progression [141]. *miR-98* was found to be downregulated in the leukemia drug-resistant cell line K562/A02, and its upregulation was shown to reduce leukemia cell proliferation and enhance chemosensitivity by inhibiting *E2F1* expression, highlighting the potential of *miR-98* in overcoming leukemia multidrug resistance [89]. High *miR-98* expression in acute myeloid leukemia (AML) patients undergoing chemotherapy was reported to be associated with longer survival outcomes, implying its prognostic value [142]. The potential utility of *miR-98*, among others, in classifying cellular or molecular subgroups of childhood acute leukemias (AL) was underscored, although specific expression details or regulatory impacts in AL were not provided [88].

The various roles of *miR-98* across leukemia subtypes highlight its potential as a therapeutic and prognostic tool, emphasizing the importance of further research into its diverse interactions within hematological malignancies.

#### Thyroid cancer (TC)

A significant portion of endocrine system tumors are thyroid cancers [89]. In thyroid carcinomas, particularly in papillary thyroid cancer (PTC), *miR-98* is often downregulated. This reduction in *miR-98* levels has been associated with more aggressive tumor phenotypes, lymph node metastasis, and a poorer prognosis. Additionally, *miR-98* seems to modulate the resistance to radioiodine therapy in advanced stages.

Researchers examined the roles and interactions of *miR-98* in papillary thyroid cancer (PTC) progression, its molecular regulation, and potential racial disparities in its expression. The regulatory mechanism wherein the lncRNA *OIP5-AS1* is targeted by *miR-98*, subsequently activating ADAMTS8 and influencing PTC cell progression, was highlighted [90]. In papillary thyroid carcinoma, a reduced *miR-98-5p* level was detailed to correspond with elevated *HMGA2* expression, which subsequently affects cell growth and apoptosis, emphasizing the regulatory role of *miR-98-5p* on *HMGA2* [143]. Racial variations in *miR-98* expression among PTC patients were spotlighted, with downregulation noted in tumor tissues compared to normal ones, suggesting potential racial implications in PTC prognosis and hinting at the possibility of tailored treatment strategies based on these differential miRNA expressions [144].

The studies emphasize the critical role of *miR-98* in influencing thyroid cancer dynamics, highlighting not only its therapeutic and prognostic potential but also the importance of considering racial disparities in its expression and subsequent treatment implications.

#### Cervical cancer (CC)

Cervical cancer is the fourth most common malignant tumor in women and the primary cause of death from reproductive tumors worldwide [145]. In cervical cancer, predominantly triggered by the human papillomavirus (HPV), there is a notable decrease in *miR-98* expression, a microRNA with implications for cell proliferation, invasion, and apoptosis.

A downregulation of *miR-98-5p* in CC tissues and cell lines was discerned, with its overexpression found to inhibit cell proliferation, invasion, migration, and EMT while promoting apoptosis. Notably, the overexpression of *miR-98-5p* was also observed to suppress the PI3K/Akt pathway in CC [90]. The significance of understanding genetic and epigenetic mechanisms in carcinogenesis, especially for cancer drug design, was underscored. In HeLa cells, a model for CC, it was highlighted that *miR-98*’s dysregulation (though unspecified as up or downregulated) could induce cell proliferation and anti-apoptosis through pathways like NFκB, TGF-β, and PI3K [93]. The role of miRNAs, including *miR-98*, in influencing drug resistance was emphasized, particularly in the case of docetaxel, even though the specific cancer type wasn’t provided [110]. High pre-*miR-98* levels across various cell lines, irrespective of malignancy or *LIN28B* expression, were reported, but its specific role in CC was not detailed. Has-*miR-98-5p* was identified as a potential but under-researched microRNA regulator in HPV oncogenesis, mainly linked to cervical and other cancers, without specifying its exact functions [146].

The studies emphasize the role of *miR-98-5p* in cervical cancer development and its influence on essential cellular pathways. Furthermore, the potential of *miR-98* in determining drug sensitivity and its relevance in HPV-related oncogenesis, especially in cervical cancer, are highlighted, warranting more detailed investigations.

#### Head and Neck squamous cell carcinoma (HNSCC)

Head and Neck Squamous Cell Carcinoma (HNSCC) is recognized as the seventh most common type of cancer globally, comprising a diverse group of tumors in the upper aerodigestive tract, with squamous cell carcinoma being the most prevalent histology [92]. In HNSCC, *miR-98* is often downregulated and is associated with tumor size, stage, and nodal metastasis. Notably, it has been implicated in mediating resistance to radiation therapy in these cancers.

In the realm of HNSCC, *miR-98* has been shown to play an important role in cancer progression and potential therapeutic pathways.

A significant decrease in *miR-98* expression in squamous cell carcinoma of the head and neck (SCCHN) was observed, with its downregulation being linked to advanced clinical stages, lymph node metastasis, and shorter survival rates. Such downregulation was found to promote malignant activities like cell proliferation, migration, and invasion through the targeting of *MTDH*, an oncogene [94]. In osteosarcoma, interactions between *miR-98-5p* and the long noncoding RNA (lncRNA) *SNHG16* were revealed, influencing cell proliferation, migration, and invasion [78]. Similar interactions were observed in oral squamous cell carcinoma (OSCC) between *miR-98-5p* and the lncRNA *HOXA11-AS* [93, 147]. NiU et al. found that *HOXA11-AS* stimulates the progression of OSCC via sponging *miR-98-5p* to upregulate the expression of *YBX2* [93]. A panel of miRNA deregulations, inclusive of *miR-98*, was highlighted in HNSCC, suggesting a potential significance of miRNA in head and neck/oral cancer progression, though specific findings about *miR-98* were not detailed [148]. In laryngeal squamous cell carcinoma (LSCC), a notable reduction of *miR-98* was documented. The overexpression of *miR-98* in this context was found to reverse epithelial-to-mesenchymal transition (EMT) and inhibit metastasis by targeting *HMGA2* []. The regulatory impact of *miR-98* on *HMGA2* expression in HNSCC under hypoxic conditions was recognized, correlating with increased chemoresistance to drugs like doxorubicin and cisplatin [149].

The diverse interactions of *miR-98* with various oncogenes and long noncoding RNAs in HNSCC indicate its significant regulatory role in disease progression and treatment outcomes. This emphasizes the importance of further exploring *miR-98*’s potential as both a therapeutic target and a key player in understanding HNSCC pathogenesis.

#### Renal cell carcinoma (RCC)

Renal cell adenocarcinoma is one of the ten most common cancers worldwide, accounting for 85% of patients with primary renal neoplasms. The mortality rate of RCC has increased to 40% due to its significant increase in frequency [150]. In RCC, the most common form of kidney cancer, *miR-98* dysregulation varies among subtypes. While it’s downregulated in clear cell RCC, pointing towards a tumor-suppressive role, in chromophobe RCC, it appears to be upregulated, suggesting context-dependent functions.

The potential of the dendritic cell vaccine DC-Ad-GM·CAIX in RCC treatment was investigated, with the therapy’s safety and effectiveness observed in Balb/c mouse models. However, tumors that evaded this immunotherapy were found to display altered immunoediting mechanisms, with differential gene expression and therapy evasion miRNAs, including *miR-98*. The specifics of *miR-98*’s regulation were not detailed [151]. In a study on clear cell renal cell carcinoma (ccRCC) subtypes, *miR-98* was identified as part of a set of miRNAs in competing gene pairs with a notable accuracy of over 92% in predicting ccRCC subtypes. Yet the exact regulatory status of *miR-98* remained unspecified [152].

#### Osteosarcoma (OS)

Osteosarcoma, an infrequent type of bone cancer, occurs at a rate of about 3.4 cases per million people each year worldwide, predominantly affecting children and teenagers [153]. In osteosarcoma, a primary bone malignancy, *miR-98* is commonly downregulated. This decreased expression promotes cell growth, migration, and invasion. Moreover, *miR-98* has been studied for its role in mediating chemotherapy resistance, particularly to drugs like cisplatin.

In the context of osteosarcoma, the involvement of lncRNA *SNHG16*, which is believed to act as a molecular sponge for *miR-98-5p* in modulating different cellular activities, has been unveiled. It has been elucidated that the effects provoked by the knockdown of *SNHG16* can be countered by inhibiting *miR-98*-5p, underscoring a significant interplay [154]. In another observation, *miR-98-5p* was presented as a pivotal hub in the miRNA-mRNA network, suggesting its essential importance in the molecular matrix of osteosarcoma. Through extensive network analysis, the potential of *miR-98-5p* and other miRNAs as likely therapeutic targets or biomarkers has been proposed, emphasizing their potential impact on diagnostic or treatment strategies for osteosarcoma [39].

#### Endometrial cancer (EC)

Endometrial cancer ranks as the sixth most common cancer among women worldwide [155]. *MiR-98*’s role in endometrial carcinomas, especially in its early stages, has garnered attention. Reduced expression of *miR-98* has been correlated with deep myometrial invasion and lymphatic metastasis, hinting at its role in tumor progression.

Transitioning to endometrial cancer, *miR-98* has been depicted as a tumor suppressor. Specifically, the role of *miR-98* in mediating paclitaxel resistance by attenuating the expression of *MRP-7* (Multidrug Resistance Protein 7) was demonstrated. A linkage between the downregulation of *miR-98* and augmented *MRP-7* levels was established, thereby promoting paclitaxel resistance and enhanced cellular invasiveness in EC [94]. Conversely, the changing expressions of *miR-98* during the evolution from normal to cancerous states in endometrial tissues were explored, showing an inverse relationship between *miR-98* and *PGRMC1* (Progesterone Receptor Membrane Component 1). Notably, an overexpression of *miR-98* was found to lead to repression of *PGRMC1* and *CYP19A1* genes, subsequently resulting in decreased cell proliferation rates in EC cells [].

#### Melanomas

Melanoma, the most lethal type of skin cancer, has been on the rise throughout the past thirty years and is the leading cause of skin cancer-related deaths worldwide [156]. It affects both male and female patients, ranking fifth and sixth among all human tumors based on current epidemiological investigations [157]. In cutaneous melanomas, *miR-98* appears to have an intricate role. Elevated levels of *miR-98* have been associated with the early stages of melanoma but are often reduced in the advanced, metastatic stages. This suggests that its role might shift from being potentially oncogenic in the early stages to tumor-suppressive in the later stages.

In studies of melanoma, a decline in *miR-98* expression was found to be correlated with progressing tumor stages and escalating metastasis. Through these investigations, the inhibitory effects of *miR-98* on melanoma cell migration and metastatic tumor size were revealed, orchestrated through a newly discovered *miR-98*-*IL-6*-negative feedback loop. This interplay between *miR-98* and *IL-6* via the Stat3-NF-κB-lin28B pathway is indicative of a complex regulatory mechanism influencing melanoma progression [96].

#### Glioblastoma Multiforme (GBM)

In glioblastoma multiforme, one of the most aggressive brain tumors, *miR-98* has been found to be downregulated. This decrease in *miR-98* levels correlates with enhanced cellular proliferation, migration, and angiogenesis. Some studies also indicate its role in mediating temozolomide resistance in GBM.

In GBM, the diagnostic and therapeutic potential of *miR-98* was emphasized. A set of serum exosomal miRNAs, including *miR-98*-5p, was highlighted as potential diagnostic biomarkers due to their differential expression compared to normal controls. These miRNAs were found to be related to cell proliferation and signaling pathways in GBM, impacting prognosis [158]. On the other hand, the downregulation of *miR-98* in glioma tissues was shown, revealing its crucial role in managing glioma cell migration and invasion. When *miR-98* was re-expressed, the invasive potential of glioma cells was reduced. The targeting of the transcription factor pre-B-cell leukemia homeobox 3 (*PBX3*) by *miR-98* further emphasized its importance in controlling GBM invasion, suggesting that the overexpression of *miR-98* might be a potential therapeutic strategy to modulate *PBX3* [97].

### *miR-98* in cancer diagnosis and prognosis

*MiR-98* has emerged as a significant player in the landscape of cancer diagnosis and prognosis, playing multiple functions across various cancer types. For instance, high expression of *miR-98* has been identified as a positive prognostic factor in acute myeloid leukemia patients undergoing chemotherapy, indicating its potential as a therapeutic target [159]. In breast cancer, *miR-98* can inhibit angiogenesis and invasion, primarily by suppressing the expression of *ALK4* and *MMP11*, emphasizing its potential utility in prognosis and treatment approaches [159].

Moreover, circulating *miR-98*, as detected in serum, exhibits promise as a biomarker for both diagnosis and prognosis in colorectal cancer, although further clarification is warranted to fully understand its clinical significance [60]. In non-small cell lung cancer (NSCLC), a reduction in serum *miR-98* levels correlates with an unfavorable prognosis, suggesting its prognostic merit. Additionally, *miR-98*’s expression level has an inverse relationship with the *TWIST* mRNA level in NSCLC, providing novel potential for understanding and fighting this malignancy [47, 55].

The investigative route to the function of *miR-98* extends to its capability of inhibiting tumor angiogenesis and invasion by targeting specific genes like activin receptor-like kinase-4 and matrix metalloproteinase-11, which further broadens our understanding of its anti-cancer attributes [159]. As scientific research explores further into the molecular complexity of *miR-98*, the potential for innovative diagnostic and prognostic tools, as well as therapeutic strategies, continues to unfold.

### Therapeutic strategies for modulating *miR-98* expression in cancer cells

Modulating *miR-98* expression in cancer cells demonstrates a variety of therapeutic strategies, given its established role in cancer progression and metastasis. For instance, a study indicated that the CCL18-mediated down-regulation of *miR-98* enhanced epithelial-to-mesenchymal transition (EMT) in breast cancer cells, promoting metastasis. This modulation was explored in hepatocellular carcinoma-conditioned tumor-associated macrophages, exhibiting the potential of *miR-98* mimics in treatment [127]. *MiR-98* displayed the potential to reduce resistance to cisplatin therapy, suggesting a therapeutic benefit in enhancing chemosensitivity [94].

Further, silencing *miR-98* expression has been observed to induce cell proliferation, migration, and invasion in nasopharyngeal carcinoma (NPC) cells, both in vitro and in vivo, indicating a potential strategy of *miR-98* inhibition for preventing cancer progression [160]. In the case of gastric cancer, lentivirus-mediated *miR-98* overexpression in gastric cancer stem cells (GCSCs) showed a potential therapeutic path, reflecting the miRNA’s impact on cancer cell stemness. Moreover, *miR-98*-mediated macrophage polarization in the progression of hepatocellular carcinoma provides another aspect of therapeutic modulation, broadening the scope of *miR-98*’s influence on the tumor microenvironment and subsequent therapeutic interventions [127].

These insights collectively demonstrate a diverse landscape of therapeutic strategies centered on *miR-98* modulation, ranging from enhancing chemosensitivity to modulating cancer cell behaviors and interacting with the tumor microenvironment, all of which offer significant promise for advancing cancer treatment approaches.

## Conclusions

The exploration of *miR-98*’s role in cancer biology has revealed its potential as an important modulator of tumoral and microenvironmental behavior. *MiR-98* has been implicated in various aspects of cancer progression, including cell proliferation, migration, and invasion, as well as chemoresistance and macrophage polarization [94, 127, 160, 161]. However, the way to understanding *miR-98*’s full therapeutic potential involves dealing with challenges and depends on a thorough understanding of its molecular and cellular interactions.

One significant challenge resides in the complex nature of *miR-98*’s molecular pathways. Its interaction with diverse molecular targets such as *ALK4, MMP11*, and *STAT3* points out a complex network of regulatory mechanisms that may exhibit variable behaviors across different cancer types and stages [159, 160]. The impact of *miR-98* modulation on the tumor microenvironment, particularly its role in macrophage polarization, further compounds the complexity and necessitates a nuanced approach to therapeutic strategy development [127].

Moreover, the delivery and stability of *miR-98* modulating agents present another layer of challenge. Effective delivery systems that ensure targeted and sustained *miR-98* modulation while minimizing off-target effects are imperative for translating preclinical findings into clinical success. Additionally, the potential for acquired resistance to *miR-98*-based therapies, similar to other molecularly targeted therapies, requires thorough investigation.

In the future, an integrated approach encompassing robust preclinical models, advanced delivery systems, and comprehensive molecular studies is vital. Uncovering the broader spectrum of *miR-98*’s interactions within the cancerous environment and its crosstalk with other regulatory molecules and pathways will be essential in developing effective therapeutic strategies. Additionally, multi-centric clinical trials evaluating the safety, efficacy, and optimal delivery methods for *miR-98* modulating agents are crucial for advancing this therapeutic frontier.

Interdisciplinary collaborations among molecular biologists, oncologists, and nanotechnology researchers could also foster innovative approaches to the issues at hand. The merging of insights from molecular studies, clinical observations, and nano-delivery platforms may pave the way towards utilizing *miR-98*’s therapeutic promise in cancer treatment, announcing a new era of targeted molecular therapies.

In conclusion, *miR-98* holds substantial promise as a candidate for cancer therapeutics. Overcoming the outlined challenges and relying on future opportunities could significantly accelerate the way toward effective *miR-98*-based cancer therapeutic strategies.

## Data Availability

No datasets were generated or analysed during the current study.

## References

[1] Pérez-Amado CJ, Bazan-Cordoba A, Hidalgo-Miranda A, Jiménez-Morales S (2021). Mitochondrial heteroplasmy shifting as a potential biomarker of cancer progression. Int J Mol Sci.

[2] Sung H, Ferlay J, Siegel RL, Laversanne M, Soerjomataram I, Jemal A (2021). Global cancer statistics 2020: GLOBOCAN estimates of incidence and mortality worldwide for 36 cancers in 185 countries. Cancer J Clin.

[3] Pajares MJ, Alemany-Cosme E, Goñi S, Bandres E, Palanca-Ballester C, Sandoval J (2021). Epigenetic regulation of microRNAs in cancer: shortening the distance from bench to bedside. Int J Mol Sci.

[4] Leite DJ, Ninova M, Hilbrant M, Arif S, Griffiths-Jones S, Ronshaugen M (2016). Pervasive microRNA duplication in chelicerates: insights from the embryonic microRNA repertoire of the spider Parasteatoda tepidariorum. Genome Biol Evol.

[5] Mazziotta C, Cervellera CF, Lanzillotti C, Touzé A, Gaboriaud P, Tognon M (2023). MicroRNA dysregulations in Merkel cell carcinoma: molecular mechanisms and clinical applications. J Med Virol.

[6] Albano GD, Gagliardo R, Montalbano AM, Profita M (2022). Non-coding RNAs in airway diseases: a brief overview of recent data. Cancers.

[7] Mohammed OA. From strings to signals: unraveling the impact of miRNAs on diagnosis, and progression of colorectal cancer. Pathology-Research Pract. 2023:154857. 10.1016/j.prp.2023.154857 37804545

[8] Elfaituri M, Khaled A (2023). The role of microRNA-1246 in early detection of breast cancer: findings from a systematic review and meta-analysis. ESMO Open.

[9] Quinn JJ, Chang HY (2016). Unique features of long non-coding RNA biogenesis and function. Nat Rev Genet.

[10] Yao R-W, Wang Y, Chen L-L (2019). Cellular functions of long noncoding RNAs. Nat Cell Biol.

[11] Zhang X, Wang S, Wang H, Cao J, Huang X, Chen Z (2019). Circular RNA circNRIP1 acts as a microRNA-149-5p sponge to promote gastric cancer progression via the AKT1/mTOR pathway. Mol Cancer.

[12] Wang X, Sun W, Shen W, Xia M, Chen C, Xiang D (2016). Long non-coding RNA DILC regulates liver cancer stem cells via IL-6/STAT3 axis. J Hepatol.

[13] Li Q, Zhou X, Zhou X (2019). Downregulation of miR–98 contributes to hypoxic pulmonary hypertension by targeting ALK1. Mol Med Rep.

[14] Yuan H, Deng R, Zhao X, Chen R, Hou G, Zhang H (2017). SUMO1 modification of KHSRP regulates tumorigenesis by preventing the TL-G-Rich miRNA biogenesis. Mol Cancer.

[15] Ha M, Kim VN (2014). Regulation of microRNA biogenesis. Nat Rev Mol Cell Biol.

[16] Suster I, Feng Y (2021). Multifaceted regulation of MicroRNA biogenesis: essential roles and functional integration in neuronal and glial development. Int J Mol Sci.

[17] Bozgeyik I (2023). miRNAs, cancer, and unconventional miRNA functions. Bull Biotechnol.

[18] Khan R, Kadamkode V, Kesharwani D, Purkayastha S, Banerjee G, Datta M (2020). Circulatory mir-98-5p levels are deregulated during diabetes and it inhibits proliferation and promotes apoptosis by targeting PPP1R15B in keratinocytes. RNA Biol.

[19] Yang G, Zhang X, Shi J (2015). MiR-98 inhibits cell proliferation and invasion of non-small cell carcinoma lung cancer by targeting PAK1. Int J Clin Exp Med.

[20] Khan R, Verma AK, Datta M. Mir-98-5p regulates gluconeogenesis and lipogenesis by targeting PPP1R15B in hepatocytes. J Cell Communication Signal. 2023:1–15. 10.1007/s12079-023-00735-0 PMC10409962 36917438

[21] Cui X, Zhang C, Wang F, Zhao X, Wang S, Liu J et al. Latexin regulates sex dimorphism in hematopoiesis via gender-specific differential expression of microRNA 98-3p and thrombospondin 1. Cell Rep. 2023;42(3). 10.1016/j.celrep.2023.112274 PMC10160986 36933218

[22] Fujii YR. Oxford miRNA Gardener: MicroRNA blossoms. The MicroRNA 2000 Transformer: Quantum Computing and Artificial Intelligence for Health. Springer; 2023. pp. 7–24.

[23] Wang Y-X, Ma J-S, Li R-N, Wang J, Lian T-Y, Zhou Y-P et al. MicroRNAs and their regulators: potential therapeutic targets in pulmonary arterial hypertension. Vascul Pharmacol. 2023:107216. 10.1016/j.vph.2023.107216 37699495

[24] Aryan L, Medzikovic L, Ruffenach G, Li M, Rahman S, Esdin L (2022). Mir98 regulates myocardial ischemia-reperfusion Injury in late pregnancy by targeting Stat3 and Pgc-1α. Circulation.

[25] Morchio M, Sher E, Collier DA, Lambert DW, Boissonade FM (2023). The role of miRNAs in Neuropathic Pain. Biomedicines.

[26] Zhao Y, Shen M, Wu L, Yang H, Yao Y, Yang Q (2023). Stromal cells in the tumor microenvironment: accomplices of tumor progression?. Cell Death Dis.

[27] Zhao L, Chang Q, Cong Z, Zhang Y, Liu Z, Zhao Y. Effects of dietary polyphenols on maternal and fetal outcomes in maternal diabetes. Food & Function; 2023. 10.1039/d3fo02048g 37724008

[28] Tregub PP, Ibrahimli I, Averchuk AS, Salmina AB, Litvitskiy PF, Manasova ZS (2023). The role of microRNAs in Epigenetic Regulation of Signaling pathways in neurological pathologies. Int J Mol Sci.

[29] Lehmann TP, Golik M, Olejnik J, Łukaszewska M, Markowska D, Drożdżyńska M (2023). Potential applications of using tissue-specific EVs in targeted therapy and vaccinology. Biomed Pharmacother.

[30] Tang S, Li S, Liu T, He Y, Hu H, Zhu Y (2021). MicroRNAs: emerging oncogenic and tumor-suppressive regulators, biomarkers and therapeutic targets in lung cancer. Cancer Lett.

[31] Yang M, Wei W (2019). SNHG16: a novel long-non coding RNA in human cancers. OncoTargets Therapy.

[32] Zhan P, Shu X, Chen M, Sun L, Yu L, Liu J (2021). Mir-98-5p inhibits gastric cancer cell stemness and chemoresistance by targeting branched-chain aminotransferases 1. Life Sci.

[33] Fei X, Zhang P, Pan Y, Liu Y (2020). MicroRNA-98-5p inhibits tumorigenesis of hepatitis B virus-related hepatocellular carcinoma by targeting NF-κB-inducing kinase. Yonsei Med J.

[34] Liu S, Zhou Y, Zhou Y, Wang J, Ji R (2020). Mechanism of miR–98 inhibiting tumor proliferation and invasion by targeting IGF1R in diabetic patients combined with colon cancer. Oncol Lett.

[35] Cai C, Huo Q, Wang X, Chen B, Yang Q. SNHG16 contributes to breast cancer cell migration by competitively binding miR-98 with E2F5. Biochem Biophys Res Commun. 2017;485(2):272–8. 10.1016/j.bbrc.2017.02.094 28232182

[36] Shi J, Ci Y, Zheng Y, Chen W, Chen X (2021). Submicron silica particles have cytotoxicities on hepatocellular carcinoma, non-small cell lung cancer and breast cancer by unified regulating the XLOC_001659/miR-98-5p/MAP3K2-mediated pathway. Toxicol Res.

[37] Li C-W, Chen B-S (2016). Investigating core genetic-and-epigenetic cell cycle networks for stemness and carcinogenic mechanisms, and cancer drug design using big database mining and genome-wide next-generation sequencing data. Cell Cycle.

[38] Mirzaei S, Gholami MH, Mahabady MK, Nabavi N, Zabolian A, Banihashemi SM (2021). Pre-clinical investigation of STAT3 pathway in bladder cancer: paving the way for clinical translation. Biomed Pharmacother.

[39] Xu K, Zhang P, Zhang J, Quan H, Wang J, Liang Y (2021). Identification of potential micro-messenger RNAs (miRNA–mRNA) interaction network of osteosarcoma. Bioengineered.

[40] Rao X, Wan L, Jie Z, Zhu X, Yin J, Cao H (2019). Upregulated miR-27a-3p indicates a poor prognosis in pancreatic carcinoma patients and promotes the angiogenesis and migration by epigenetic silencing of GATA6 and activating VEGFA/VEGFR2 signaling pathway. OncoTargets Therapy.

[41] Ghoncheh M, Pournamdar Z, Salehiniya H (2016). Incidence and mortality and epidemiology of breast cancer in the world. Asian Pac J Cancer Prev.

[42] Li C, Li Z, Yi H, Liu Z (2022). IncRNA Linc00511 Upregulation elevates TGFBR1 and participates in the postoperative distant recurrence of Non-small-cell Lung Cancer by Targeting miR-98-5p. Crit Rev Eukaryot Gene Expr.

[43] Jiang F, Yu Q, Chu Y, Zhu X, Lu W, Liu Q (2019). MicroRNA-98-5p inhibits proliferation and metastasis in non-small cell lung cancer by targeting TGFBR1. Int J Oncol.

[44] Giaquinto AN, Sung H, Miller KD, Kramer JL, Newman LA, Minihan A (2022). Breast Cancer Stat 2022 CA: cancer J Clin.

[45] Liu H, Ye H (2017). Screening of the prognostic targets for breast cancer based co-expression modules analysis. Mol Med Rep.

[46] Guo C, Zhang M, Qian J, Li P, Guo L (2022). Oncogenic long noncoding RNA Linc01287 promotes IGF1R expression by sponging miR-98 in breast Cancer. Crit Rev Eukaryot Gene Expr.

[47] Sun D, Luo X, Ma L, Wang Y, Zhang F (2020). Identifying of miR-98-5p/IGF1 axis contributes breast cancer progression using comprehensive bioinformatic analyses methods and experiments validation. Life Sci.

[48] Zhang M, Li Z, Liu X (2021). MiR-98-5p/IGF2 Axis Influence Herceptin sensitivity through IGF1R/HER2 heterodimer formation and AKT/mTOR Signal Pathway in HER2 positive breast Cancer. Asian Pac J cancer Prevention: APJCP.

[49] Liu W, Xiao P, Wu H, Wang L, Kong D, Yu F (2017). MicroRNA-98 plays a suppressive role in Non-small Cell Lung Cancer through inhibition of SALL4 protein expression. Oncol Res.

[50] Cai C, Huo Q, Wang X, Chen B, Yang Q (2017). SNHG16 contributes to breast cancer cell migration by competitively binding miR-98 with E2F5. Biochem Biophys Res Commun.

[51] Jiang P, Wu X, Wang X, Huang W, Feng Q (2016). NEAT1 upregulates EGCG-induced CTR1 to enhance cisplatin sensitivity in lung cancer cells. Oncotarget.

[52] Zhu M, Zhang C, Chen D, Chen S, Zheng H (2019). MicroRNA-98-HMGA2-POSTN signal pathway reverses epithelial-to-mesenchymal transition in laryngeal squamous cell carcinoma. Biomed Pharmacotherapy = Biomedecine Pharmacotherapie.

[53] Xiang Q, Tang H, Yu J, Yin J, Yang X, Lei X (2013). MicroRNA-98 sensitizes cisplatin-resistant human lung adenocarcinoma cells by up-regulation of HMGA2. Pharmazie.

[54] Du L, Schageman JJ, Subauste MC, Saber B, Hammond SM, Prudkin L (2009). miR-93, miR-98, and miR-197 regulate expression of tumor suppressor gene FUS1. Mol cancer Research: MCR.

[55] Liu W-L, Chang J-M, Chong I-W, Hung Y-L, Chen Y-H, Huang W-T, et al. Curcumin inhibits LIN-28A through the activation of miRNA-98 in the lung cancer cell line A549. Molecules. 2017;22(6):929. 10.3390/molecules22060929 PMC6152786 28587210

[56] Zhou DH, Wang X, Feng Q (2014). EGCG enhances the efficacy of cisplatin by downregulating hsa-mir-98-5p in NSCLC A549 cells. Nutr Cancer.

[57] Ye J, Yan Y, Xin L, Liu J, Tang T, Bao X (2022). Long non-coding RNA TMPO-AS1 facilitates the progression of colorectal cancer cells via sponging mir-98-5p to upregulate BCAT1 expression. J Gastroenterol Hepatol.

[58] Kenneth MJ, Shishir TA, Haque FKM (2023). In silico analysis reveals mir-98-5p as a potential inhibitor of tumor cell proliferation and metastasis in colorectal cancer by targeting the fzd3 receptor of the wnt signaling pathway. J Genetic Eng Biotechnol.

[59] Fu Q, Cheng J, Zhang J, Zhang Y, Chen X, Xie J, et al. Periostin regulated by let-7/miR-98 family mediates the apoptosis and epithelial-mesenchymal transition of colon cancer. Zhonghua Zhong liu za zhi [Chin J Oncol]. 2019;41(8):573–9. 10.3760/cma.j.issn.0253-3766.2019.08.004 31434447

[60] Zheng YF, Luo J, Gan GL, Li W (2019). Overexpression of microRNA-98 inhibits cell proliferation and promotes cell apoptosis via claudin-1 in human colorectal carcinoma. J Cell Biochem.

[61] Milanesi E, Dobre M, Bucuroiu AI, Herlea V, Manuc TE, Salvi A (2020). miRNAs-Based molecular signature for KRAS Mutated and Wild Type Colorectal Cancer: an explorative study. J Immunol Res.

[62] Pathak S, Meng WJ, Nandy SK, Ping J, Bisgin A, Helmfors L (2015). Radiation and SN38 treatments modulate the expression of microRNAs, cytokines and chemokines in colon cancer cells in a p53-directed manner. Oncotarget.

[63] Zhang JJ, Chen JT, Hua L, Yao KH, Wang CY (2017). miR-98 inhibits hepatocellular carcinoma cell proliferation via targeting EZH2 and suppressing Wnt/β-catenin signaling pathway. Biomed Pharmacotherapy = Biomedecine Pharmacotherapie.

[64] Yahya SMM, Yahya SMM (2020). The effect of miR-98 and miR-214 on apoptotic and angiogenic pathways in Hepatocellular Carcinoma HepG2 cells. Indian J Clin Biochemistry: IJCB.

[65] Ji PT, Wang XY (2023). Clinical application study on mir-98-5p as a prognostic biomarker in hepatocellular carcinoma. Clin Res Hepatol Gastroenterol.

[66] Shi J, Ci Y, Zheng Y, Chen W, Chen X. Submicron silica particles have cytotoxicities on hepatocellular carcinoma, non-small cell lung cancer and breast cancer by unified regulating the XLOC_001659/miR-98-5p/MAP3K2-mediated pathway. Toxicol Res. 2021;10(4):824–34. 10.1093/toxres/tfab062 PMC8403595 34484674

[67] Jiang T, Li M, Li Q, Guo Z, Sun X, Zhang X (2017). MicroRNA-98-5p inhibits cell proliferation and induces cell apoptosis in Hepatocellular Carcinoma via Targeting IGF2BP1. Oncol Res.

[68] Zhou W, Zou B, Liu L, Cui K, Gao J, Yuan S (2016). MicroRNA-98 acts as a tumor suppressor in hepatocellular carcinoma via targeting SALL4. Oncotarget.

[69] Gan H, Lin L, Hu N, Yang Y, Gao Y, Pei Y (2019). Aspirin ameliorates lung cancer by targeting the miR-98/WNT1 axis. Thorac cancer.

[70] Hou W, Tian Q, Steuerwald NM, Schrum LW, Bonkovsky HL (2012). The let-7 microRNA enhances heme oxygenase-1 by suppressing Bach1 and attenuates oxidant injury in human hepatocytes. Biochim Biophys Acta.

[71] Guo Z, He C, Yang F, Qin L, Lu X, Wu J. Long non-coding RNA-NEAT1, a sponge for miR-98-5p, promotes expression of oncogene HMGA2 in prostate cancer. Biosci Rep. 2019;39(9):BSR20190635. 10.1042/BSR20190635 PMC6757183 31481527

[72] Yahya SMM, Elmegeed GA, Mohamed MS, Mohareb RM, Abd-Elhalim MM, Elsayed GH (2018). The Effect of newly synthesized heterosteroids on miRNA34a, 98, and 214 expression levels in MCF-7 breast Cancer cells. Indian J Clin Biochemistry: IJCB.

[73] Wan L, Thomas-Ahner JM, Pearl DK, Erdman JW, Moran NE, Clinton SK (2023). Orchestration of miRNA patterns by Testosterone and Dietary Tomato carotenoids during early prostate carcinogenesis in TRAMP mice. J Nutr.

[74] Fu Y, Liu X, Chen Q, Liu T, Lu C, Yu J (2018). Downregulated mir-98-5p promotes PDAC proliferation and metastasis by reversely regulating MAP4K4. J Experimental Clin cancer Research: CR.

[75] Siragam V, Rutnam ZJ, Yang W, Fang L, Luo L, Yang X (2012). MicroRNA miR-98 inhibits tumor angiogenesis and invasion by targeting activin receptor-like kinase-4 and matrix metalloproteinase-11. Oncotarget.

[76] Xu QF, Peng HP, Lu XR, Hu Y, Xu ZH, Xu JK (2021). Oleanolic acid regulates the Treg/Th17 imbalance in gastric cancer by targeting IL-6 with miR-98-5p. Cytokine.

[77] Yan LR, Ding HX, Shen SX, Lu XD, Yuan Y, Xu Q (2021). Pepsinogen C expression-related lncRNA/circRNA/mRNA profile and its co-mediated ceRNA network in gastric cancer. Funct Integr Genom.

[78] Deng ZQ, Yin JY, Tang Q, Liu FQ, Qian J, Lin J (2014). Over-expression of miR-98 in FFPE tissues might serve as a valuable source for biomarker discovery in breast cancer patients. Int J Clin Exp Pathol.

[79] Wang Y, Bao W, Liu Y, Wang S, Xu S, Li X (2018). Mir-98-5p contributes to cisplatin resistance in epithelial ovarian cancer by suppressing miR-152 biogenesis via targeting Dicer1. Cell Death Dis.

[80] Guo H, Ha C, Dong H, Yang Z, Ma Y, Ding Y (2019). Cancer-associated fibroblast-derived exosomal microRNA-98-5p promotes cisplatin resistance in ovarian cancer by targeting CDKN1A. Cancer Cell Int.

[81] Wendler A, Keller D, Albrecht C, Peluso JJ, Wehling M (2011). Involvement of let-7/miR-98 microRNAs in the regulation of progesterone receptor membrane component 1 expression in ovarian cancer cells. Oncol Rep.

[82] Liu C, Zhao S, Lv ZX, Zhao XJ (2023). Promoting action of long non-coding RNA small nucleolar RNA host gene 4 in ovarian cancer. Acta Biochim Pol.

[83] Luo H, Yang L, Liu C, Wang X, Dong Q, Liu L (2020). TMPO-AS1/miR-98-5p/EBF1 feedback loop contributes to the progression of bladder cancer. Int J Biochem Cell Biol.

[84] Xia Z, Wang Q, Lu P (2023). LncRNA LINC00885 promotes bladder cancer progression by targeting the miR-98-5p/PBX3 axis. Cellular and molecular biology. France).

[85] Luan T, Fu S, Huang L, Zuo Y, Ding M, Li N (2018). MicroRNA-98 promotes drug resistance and regulates mitochondrial dynamics by targeting LASS2 in bladder cancer cells. Exp Cell Res.

[86] Feng F, Chen A, Huang J, Xia Q, Chen Y, Jin X (2018). Long noncoding RNA SNHG16 contributes to the development of bladder cancer via regulating miR-98/STAT3/Wnt/β-catenin pathway axis. J Cell Biochem.

[87] Huang Y, Hong X, Hu J, Lu Q (2017). Targeted regulation of MiR-98 on E2F1 increases chemosensitivity of leukemia cells K562/A02. OncoTargets Therapy.

[88] Zhang X, Li D, Jia C, Cai H, Lv Z, Wu B (2021). METTL14 promotes tumorigenesis by regulating lncRNA OIP5-AS1/miR-98/ADAMTS8 signaling in papillary thyroid cancer. Cell Death Dis.

[89] Qiu K, Xie Q, Jiang S, Lin T (2020). Mir-98-5p promotes apoptosis and inhibits migration and cell growth in papillary thyroid carcinoma through Bax/Caspase-3 by HMGA2. J Clin Lab Anal.

[90] Okuno J, Miyake T, Sota Y, Tanei T, Kagara N, Naoi Y (2021). Development of Prediction Model Including MicroRNA expression for Sentinel Lymph Node Metastasis in ER-Positive and HER2-Negative breast Cancer. Ann Surg Oncol.

[91] Zhang M, Guo C, Chu Y, Xu R, Yin F, Qian J (2022). [Dihydromyricetin reverses herceptin resistance by up-regulating mir-98-5p and inhibiting IGF1R/HER2 dimer formation in SKBR3 cells]. Nan Fang Yi Ke da xue xue bao = Journal. South Med Univ.

[92] Tan H, Zhu G, She L, Wei M, Wang Y, Pi L (2017). MiR-98 inhibits malignant progression via targeting MTDH in squamous cell carcinoma of the head and neck. Am J Cancer Res.

[93] Triantafyllou A, Dovrolis N, Zografos E, Theodoropoulos C, Zografos GC, Michalopoulos NV (2022). Circulating miRNA expression profiling in breast Cancer Molecular subtypes: applying machine learning analysis in Bioinformatics. Cancer Diagnosis Prognosis.

[94] Huang W, Zhang J, Dong B, Chen H, Shao L, Li X (2021). A novel miR-98 negatively regulates the resistance of Endometrial Cancer cells to Paclitaxel by suppressing ABCC10/MRP-7. Front Oncol.

[95] Panda H, Chuang TD, Luo X, Chegini N (2012). Endometrial miR-181a and miR-98 expression is altered during transition from normal into cancerous state and target PGR, PGRMC1, CYP19A1, DDX3X, and TIMP3. J Clin Endocrinol Metab.

[96] Li F, Li XJ, Qiao L, Shi F, Liu W, Li Y (2014). miR-98 suppresses melanoma metastasis through a negative feedback loop with its target gene IL-6. Exp Mol Med.

[97] Xu X, Bao Z, Liu Y, Ji J, Liu N (2017). MicroRNA-98 attenuates Cell Migration and Invasion in Glioma by directly targeting Pre-B Cell Leukemia Homeobox 3. Cell Mol Neurobiol.

[98] Kim S (2018). Computational model for Predicting the Relationship between Micro-RNAs and their target Messenger RNAs in breast and Colon cancers. Cancer Inform.

[99] Farazi TA, Horlings HM, Ten Hoeve JJ, Mihailovic A, Halfwerk H, Morozov P (2011). MicroRNA sequence and expression analysis in breast tumors by deep sequencing. Cancer Res.

[100] Geretto M, Pulliero A, Rosano C, Zhabayeva D, Bersimbaev R, Izzotti A (2017). Resistance to cancer chemotherapeutic drugs is determined by pivotal microRNA regulators. Am J Cancer Res.

[101] Bhat-Nakshatri P, Wang G, Collins NR, Thomson MJ, Geistlinger TR, Carroll JS (2009). Estradiol-regulated microRNAs control estradiol response in breast cancer cells. Nucleic Acids Res.

[102] Le DH (2015). Network-based ranking methods for prediction of novel disease associated microRNAs. Comput Biol Chem.

[103] Zhang P, Shao G, Lin X, Liu Y, Yang Z (2017). MiR-338-3p inhibits the growth and invasion of non-small cell lung cancer cells by targeting IRS2. Am J cancer Res.

[104] Fiteni F, Anota A, Westeel V, Bonnetain F (2016). Methodology of health-related quality of life analysis in phase III advanced non-small-cell lung cancer clinical trials: a critical review. BMC Cancer.

[105] Yang G, Zhang X, Shi J (2015). MiR-98 inhibits cell proliferation and invasion of non-small cell carcinoma lung cancer by targeting PAK1. Int J Clin Exp Med.

[106] Ke SB, Qiu H, Chen JM, Shi W, Han C, Gong Y (2020). ALG3 contributes to the malignancy of non-small cell lung cancer and is negatively regulated by MiR-98-5p. Pathol Res Pract.

[107] Wu F, Mo Q, Wan X, Dan J, Hu H (2019). NEAT1/hsa-mir-98-5p/MAPK6 axis is involved in non–small-cell lung cancer development. J Cell Biochem.

[108] Triantafyllou A, Dovrolis N, Zografos E, Theodoropoulos C, Zografos GC, Michalopoulos NV, et al. Circulating miRNA expression profiling in breast cancer molecular subtypes: applying machine learning analysis in bioinformatics. Cancer Diagnosis Prognosis. 2022;2(6):739–49. 10.21873/cdp.10169 PMC9628143 36340453

[109] Qin C, Lu R, Yuan M, Zhao R, Zhou H, Fan X (2021). Circular RNA 0006349 augments glycolysis and Malignance of Non-small Cell Lung Cancer cells through the microRNA-98/MKP1 Axis. Front cell Dev Biology.

[110] Liu L, Zhang Q, Peng H (2023). Circ_0048856 competes with ABCC1 for miR-193a-5p/miR-98-5p binding sites to promote the cisplatin resistance and tumorigenesis in lung cancer. J Chemother.

[111] Wang K, Dong L, Fang Q, Xia H, Hou X (2017). Low serum miR-98 as an unfavorable prognostic biomarker in patients with non-small cell lung cancer. Cancer Biomark A.

[112] Wang X, Zhang G, Cheng Z, Dai L, Jia L, Jing X (2020). Knockdown of lncRNA ANRIL inhibits the development of cisplatin resistance by upregulating miR–98 in lung cancer cells. Oncol Rep.

[113] Tang Y, Wu L, Zhao M, Zhao G, Mao S, Wang L (2019). LncRNA SNHG4 promotes the proliferation, migration, invasiveness, and epithelial–mesenchymal transition of lung cancer cells by regulating miR-98-5p. Biochem Cell Biol.

[114] Huang TH, Wu ATH, Cheng TS, Lin KT, Lai CJ, Hsieh HW (2019). In silico identification of thiostrepton as an inhibitor of cancer stem cell growth and an enhancer for chemotherapy in non-small-cell lung cancer. J Cell Mol Med.

[115] Chen L, Cao P, Huang C, Wu Q, Chen S, Chen F (2020). Serum exosomal miR-7977 as a novel biomarker for lung adenocarcinoma. J Cell Biochem.

[116] Chen X, Xu Y, Liao X, Liao R, Zhang L, Niu K (2016). Plasma miRNAs in predicting radiosensitivity in non-small cell lung cancer. Tumour Biology: J Int Soc Oncodevelopmental Biology Med.

[117] Song R, Liu Q, Hutvagner G, Nguyen H, Ramamohanarao K, Wong L (2014). Rule discovery and distance separation to detect reliable miRNA biomarkers for the diagnosis of lung squamous cell carcinoma. BMC Genomics.

[118] Wang YG, He Q, Guo SQ, Shi ZZ (2019). Reduced serum miR-98 predicts unfavorable clinical outcome of colorectal cancer. Eur Rev Med Pharmacol Sci.

[119] Zhu W, Huang Y, Pan Q, Xiang P, Xie N, Yu H (2017). MicroRNA-98 suppress Warburg Effect by Targeting HK2 in Colon cancer cells. Dig Dis Sci.

[120] Stachowiak M, Flisikowska T, Bauersachs S, Perleberg C, Pausch H, Switonski M (2017). Altered microRNA profiles during early colon adenoma progression in a porcine model of familial adenomatous polyposis. Oncotarget.

[121] Neerincx M, Poel D, Sie DLS, van Grieken NCT, Shankaraiah RC, van der Wolf-de Lijster FSW (2018). Combination of a six microRNA expression profile with four clinicopathological factors for response prediction of systemic treatment in patients with advanced colorectal cancer. PLoS ONE.

[122] Ji H, Chen M, Greening DW, He W, Rai A, Zhang W (2014). Deep sequencing of RNA from three different extracellular vesicle (EV) subtypes released from the human LIM1863 colon cancer cell line uncovers distinct miRNA-enrichment signatures. PLoS ONE.

[123] McGlynn KA, Petrick JL, El-Serag HB (2021). Epidemiology of Hepatocellular Carcinoma. Hepatology (Baltimore MD).

[124] Shen Q, Jiang S, Wu M, Zhang L, Su X, Zhao D (2020). LncRNA HEIH confers cell Sorafenib Resistance in Hepatocellular Carcinoma by regulating miR-98-5p/PI3K/AKT pathway. Cancer Manage Res.

[125] Li L, Sun P, Zhang C, Li Z, Cui K, Zhou W (2018). MiR-98 modulates macrophage polarization and suppresses the effects of tumor-associated macrophages on promoting invasion and epithelial-mesenchymal transition of hepatocellular carcinoma. Cancer Cell Int.

[126] Wang G, Dong F, Xu Z, Sharma S, Hu X, Chen D (2017). MicroRNA profile in HBV-induced infection and hepatocellular carcinoma. BMC Cancer.

[127] Fei X, Zhang P, Pan Y, Liu Y (2020). MicroRNA-98-5p inhibits tumorigenesis of Hepatitis B Virus-Related Hepatocellular Carcinoma by targeting NF-κB-Inducing kinase. Yonsei Med J.

[128] Dong L, Cao X, Luo Y, Zhang G, Zhang D (2020). A positive Feedback Loop of lncRNA DSCR8/miR-98-5p/STAT3/HIF-1α plays a role in the progression of Ovarian Cancer. Front Oncol.

[129] Wang YC, Chen YL, Yuan RH, Pan HW, Yang WC, Hsu HC, et al. Lin-28B expression promotes transformation and invasion in human hepatocellular carcinoma. Carcinogenesis. 2010;31(9):1516–22. 10.1093/carcin/bgq107 20525879

[130] Angileri F, Morrow G, Scoazec JY, Gadot N, Roy V, Huang S (2016). Identification of circulating microRNAs during the liver neoplastic process in a murine model of hereditary tyrosinemia type 1. Sci Rep.

[131] Bray F, Ferlay J, Soerjomataram I, Siegel RL, Torre LA, Jemal A (2018). Global cancer statistics 2018: GLOBOCAN estimates of incidence and mortality worldwide for 36 cancers in 185 countries. Cancer J Clin.

[132] Tsai YS, Yeh ML, Tsai PC, Huang CI, Huang CF, Hsieh MH, et al. Clusters of circulating let-7 family tumor suppressors are associated with clinical characteristics of chronic hepatitis C. Int J Mol Sci. 2020;21(14). 10.3390/ijms21144945 PMC7404305 32668728

[133] Matin F, Jeet V, Moya L, Selth LA, Chambers S, Clements JA (2018). A plasma Biomarker Panel of four MicroRNAs for the diagnosis of prostate Cancer. Sci Rep.

[134] Pashaei E, Pashaei E, Ahmady M, Ozen M, Aydin N (2017). Meta-analysis of miRNA expression profiles for prostate cancer recurrence following radical prostatectomy. PLoS ONE.

[135] Yin Y, Li M, Li H, Jiang Y, Cao LY, Zhang HF (2010). [Expressions of 6 microRNAs in prostate cancer]. Zhonghua Nan Ke xue = National. J Androl.

[136] Giglio S, De Nunzio C, Cirombella R, Stoppacciaro A, Faruq O, Volinia S, et al. A preliminary study of micro-RNAs as minimally invasive biomarkers for the diagnosis of prostate cancer patients. J Exp Clin Cancer Res: CR. 2021;40(1):79. 10.1186/s13046-021-01875-0 PMC7903618 33622375

[137] Zhang D, Fan D (2010). New insights into the mechanisms of gastric cancer multidrug resistance and future perspectives. Future Oncol.

[138] Ma Z, Liu G, Hao S, Zhao T, Chang W, Wang J (2022). PITPNA-AS1/miR-98-5p to mediate the Cisplatin Resistance of Gastric Cancer. J Oncol.

[139] Barani M, Bilal M, Sabir F, Rahdar A, Kyzas GZ (2021). Nanotechnology in ovarian cancer: diagnosis and treatment. Life Sci.

[140] Qi X, Yu C, Wang Y, Lin Y, Shen B (2019). Network vulnerability-based and knowledge-guided identification of microRNA biomarkers indicating platinum resistance in high-grade serous ovarian cancer. Clin Translational Med.

[141] Hu N, Cheng Z, Pang Y, Zhao H, Chen L, Wang C (2019). High expression of MiR-98 is a good prognostic factor in acute myeloid leukemia patients treated with chemotherapy alone. J Cancer.

[142] Hu N, Cheng Z, Pang Y, Zhao H, Chen L, Wang C, et al. High expression of MiR-98 is a good prognostic factor in acute myeloid leukemia patients treated with chemotherapy alone. J Cancer. 2019;10(1):178–85. 10.7150/jca.26391 PMC6329859 30662538

[143] Qiu K, Xie Q, Jiang S, Lin T. miR-98-5p promotes apoptosis and inhibits migration and cell growth in papillary thyroid carcinoma through Bax/Caspase-3 by HMGA2. J Clin Lab Anal. 2020;34(2):e23044. 10.1002/jcla.23044 PMC7031561 31670857

[144] Xiao R, Wang H, Yang B (2021). MicroRNA-98-5p modulates cervical cancer progression via controlling PI3K/AKT pathway. Bioengineered.

[145] Li CW, Chen BS (2016). Investigating core genetic-and-epigenetic cell cycle networks for stemness and carcinogenic mechanisms, and cancer drug design using big database mining and genome-wide next-generation sequencing data. Cell Cycle (Georgetown Tex).

[146] Mody MD, Rocco JW, Yom SS, Haddad RI, Saba NF (2021). Head and neck cancer. Lancet.

[147] Niu X, Yang B, Liu F, Fang Q (2020). LncRNA HOXA11-AS promotes OSCC progression by sponging mir-98-5p to upregulate YBX2 expression. Biomed Pharmacother.

[148] Chen D, Cabay RJ, Jin Y, Wang A, Lu Y, Shah-Khan M (2013). MicroRNA deregulations in Head and Neck squamous cell carcinomas. J oral Maxillofacial Res.

[149] Hebert C, Norris K, Scheper MA, Nikitakis N, Sauk JJ (2007). High mobility group A2 is a target for miRNA-98 in head and neck squamous cell carcinoma. Mol Cancer.

[150] Siegel RL, Miller KD, Jemal A (2019). Cancer statistics, 2019. Cancer J Clin.

[151] Birkhäuser FD, Koya RC, Neufeld C, Rampersaud EN, Lu X, Micewicz ED et al. Dendritic cell-based immunotherapy in prevention and treatment of renal cell carcinoma: efficacy, safety, and activity of Ad-GM·CAIX in immunocompetent mouse models. J Immunotherapy (Hagerstown, Md: 1997). 2013;36(2):102–11. 10.1097/CJI.0b013e31827bec97 PMC6410717 23377663

[152] Wu Y, Han W, Xu D, Wang X, Yang J, Lu Z (2021). Identification of subtype specific biomarkers of clear cell renal cell carcinoma using random forest and greedy algorithm. Bio Syst.

[153] Misaghi A, Goldin A, Awad M, Kulidjian AA (2018). Osteosarcoma: a comprehensive review. Sicot-j.

[154] Liao S, Xing S, Ma Y (2019). LncRNA SNHG16 sponges mir-98-5p to regulate cellular processes in osteosarcoma. Cancer Chemother Pharmacol.

[155] Ozdemir CY, Telli EU, Oge T, Yalcin OT (2023). Ultrasonography, macroscopy, and frozen section: whıch is better for predicting deep myometrial invasıon in endometrial cancer? Revista Da Associação Médica. Brasileira.

[156] Villanueva J, Herlyn M (2008). Melanoma and the tumor microenvironment. Curr Oncol Rep.

[157] Wahid M, Jawed A, Mandal RK, Dar SA, Akhter N, Somvanshi P (2018). Recent developments and obstacles in the treatment of melanoma with BRAF and MEK inhibitors. Crit Rev Oncol/Hematol.

[158] Yang Q, Wei B, Peng C, Wang L, Li C (2022). Identification of serum exosomal miR-98-5p, miR-183-5p, mir-323-3p and miR-19b-3p as potential biomarkers for glioblastoma patients and investigation of their mechanisms. Curr Res Translational Med.

[159] Hu N, Cheng Z, Pang Y, Zhao H, Chen L, Wang C (2019). High expression of MiR-98 is a good prognostic factor in acute myeloid leukemia patients treated with chemotherapy alone. J Cancer.

[160] Liu J, Chen W, Chen Z, Wen J, Yu H, Wang F (2017). The effects of microRNA-98 inhibits cell proliferation and invasion by targeting STAT3 in nasopharyngeal carcinoma. Biomed Pharmacotherapy = Biomedecine Pharmacotherapie.

[161] Chou CH, Shrestha S, Yang CD, Chang NW, Lin YL, Liao KW (2018). miRTarBase update 2018: a resource for experimentally validated microRNA-target interactions. Nucleic Acids Res.

